# Reservoir sedimentation diminishes water storage and coastal delta resiliency

**DOI:** 10.1038/s41467-026-76986-3

**Published:** 2026-08-25

**Authors:** Abigail C. Eckland, Melissa A. Foster, Aaron A. Hurst, Mussie T. Beyene, Irina Overeem

**Affiliations:** 1 Department of Earth and Environmental Sciences, Louisiana State University New Orleans, New Orleans, LA USA; 2 Department of Geological Sciences and Institute of Arctic and Alpine Research, University of Colorado Boulder, Boulder, CO USA; 3 Technical Service Center, Bureau of Reclamation, Denver, CO USA; 4 Department of Civil and Environmental Engineering and Utah Water Research Laboratory, Utah State University, Logan, UT USA

**Keywords:** Environmental sciences, Civil engineering, Hydrology

## Abstract

Surface water storage in dammed reservoirs diminishes over time due to sediment accumulation. Here, we applied the RATTES model to predict storage loss and sedimentation at 57,117 reservoirs across the continental United States (1700–2050), resulting in cumulative reservoir sediment volumes of 63.8 km^3^ by 2025 and 83.0 km^3^ by 2050. Although cumulative storage loss is 7.7%, over two-fifths of reservoirs have lost more than 25% of designed storage capacity. The equivalent sediment mass is 61.3 Gt, exceeding the annual global fluvial sediment load by a factor of seven. If coastal deltas received the annual sediment load currently stored in reservoirs, delta aggradation rates would be comparable to eustatic sea-level rise at half of US deltas. While 79% of reservoir sediment is stored over 500 km from the coast, 7% is sequestered behind terminal dams where sustainable sediment management could reconnect sediment transport to coasts.

## Abstract (web summary)

This study applies the RATTES model to predict storage loss and sedimentation across 57,117 reservoirs in the continental US from 1700 to 2050, quantifying accumulated reservoir sediment volumes and assessing their implications for downstream sediment delivery and coastal delta aggradation.

## Introduction

Dams impound surface water reservoirs that also trap sediment transported from upstream basins. Sediment accumulation within reservoirs over time reduces their capacity to store water for irrigation, flood control, hydropower, water supply, and recreation, and can ultimately threaten dam operations and water security^1–5^. Reservoir sedimentation also diminishes sediment fluxes to downstream reaches and coastal deltas. Sediment delivery to deltas has declined^2,3,6^ by as much as 39% relative to pre-dam conditions^7^, starving many of the world’s largest and most populated deltas of sediment^8^. Many deltas, including the United States’ Mississippi River Delta, rely on fluvial sediment to counter subsidence, erosion, and sea-level rise (SLR)^9–12^. Nevertheless, projects that augment fluxes to coasts, such as dam removal or sediment bypassing, remain rare^13^, while many more global dams are under construction or planned^14^.

Sediment trapping in reservoirs decreases water supply and starves downstream delta systems^15^; despite these potential impacts, sediment sequestration by reservoirs remains poorly constrained. These uncertainties stem from (1) the complex sequence of dam emplacement on stream networks through time, which impacts sediment routing and trapping, and (2) the historical focus on sedimentation in large reservoirs, which has overlooked tens of thousands of small headwater dams that intercept substantial sediment upstream^4,16–19^. These gaps previously limited the ability to predict remaining storage capacity for critical water services and to assess the fate of coastal deltas under future change.

To address these uncertainties and data gaps, we create the RATTES model to predict past and future water storage capacity and sediment accumulation at 57,117 reservoirs across the contiguous United States (hereafter US) from 1700 to 2050. RATTES integrates a reservoir network dataset, ResNet^20^, a living dataset of US reservoirs linked to National Hydrography Dataset flowlines (NHDPlusV2)^21^. ResNet includes 44,766 headwater dams that function as important sediment traps by disconnecting upstream sediment-contributing drainage areas from larger downstream reservoirs. Using ResNet and measured sedimentation survey data from 904 reservoirs (Supplementary Data 1), we numerically model capacity and sediment storage volumes as dams are constructed or removed, explicitly tracking changes to the sediment-contributing drainage area $({A}_{{sed}})$, which is the portion of drainage area that remains unregulated by dams. ${A}_{{sed}}$ is a key predictor of reservoir sedimentation shown to perform well in previous empirical studies^16,22–25^. Because the training data document the total volume of sediment deposited within reservoirs, RATTES estimates long-term basin sediment yields and cumulative sediment storage rather than sediment fluxes within discrete river reaches. Accordingly, the model does not represent point-source sediment contributions from tributaries, landslides, river incision, or sediment transport time lags^26–28^. Notably, 357 of the surveyed sites are headwater dams, typically excluded in previous reservoir sedimentation studies^17,18^, enabling representation of sediment trapping across the majority of US water storage infrastructure, not just at large facilities.

Sedimentation within a reservoir is partly controlled by its dam trapping efficiency, defined as the proportion of incoming river sediment that is deposited and retained within the reservoir^29,30^. Trap efficiency ranges from 0—where no sediment is trapped and all sediment is passed downstream—to 1 (100%)—where all incoming sediment is trapped. Existing trap efficiency formulas incorporate parameters such as grain size, inflow, particle setting velocity, flow velocity, drainage area, and total reservoir capacity^29–32^. A common relationship across most equations is that reductions in reservoir capacity result in lower trap efficiency^29,30,33,34^. This implies that reservoirs retain a smaller fraction of incoming sediment as (1) water residence time decreases and (2) sediment infilling progresses with age.

Simulating evolving reservoir capacity and trap efficiency across a nationwide, linked dam network is key to quantify sediment supply deficits to downstream coasts and deltas. Changing trap efficiency and capacity at upstream dams modulates the supply of sediment to downstream rivers and dams^16^. To employ a consistent methodology across all 57,117 modeled reservoirs, we use the classic Brown trap efficiency equation^29^ since it is applicable on a large scale, requiring only reservoir capacity, total drainage area, and estimated sediment grain size. We acquire capacity and drainage area from ResNet^20^ and assume silt-sized sediment, consistent with sediment-budget design assumptions^35,36^ and past studies^23,24^. After finalizing the RATTES model, we explore two endmember scenarios assuming either sand or clay-sized sediment across all modeled reservoirs. We also compare trap efficiencies calculated using the Brown^29^ and Brune^30^ equations for reservoirs with annual inflow data available (*n* = 298) (Supplementary Methods 1).

Here, we use the modeled reservoir sediment storage to consider its downstream implications for coastal deltas and to explore opportunities for reinstating sediment transport. We compare the cumulative reservoir sediment volume and sedimentation rates to demonstrate the potential magnitude of delta sediment aggradation rates that could be attained in the absence of upstream dams and, further, to evaluate how these rates compare in magnitude with eustatic SLR (mm yr^−1^). We also assess the fraction of sediment stored within terminal dams—the most downstream structures within the river network that exert the final control on sediment delivery to coastal zones—to highlight where sediment management or dam removal would most effectively restore sediment transport to deltas. In this work, we quantify the volume of reservoir sediment potentially withheld from downstream environments, convert this volume to a comparable metric (delta aggradation rates), and identify reservoir sedimentation sites that could serve as potential sediment resources for construction or restoration projects.

## Results

### RATTES: a transferable modeling approach to predict reservoir sedimentation

**Fig. 1:**
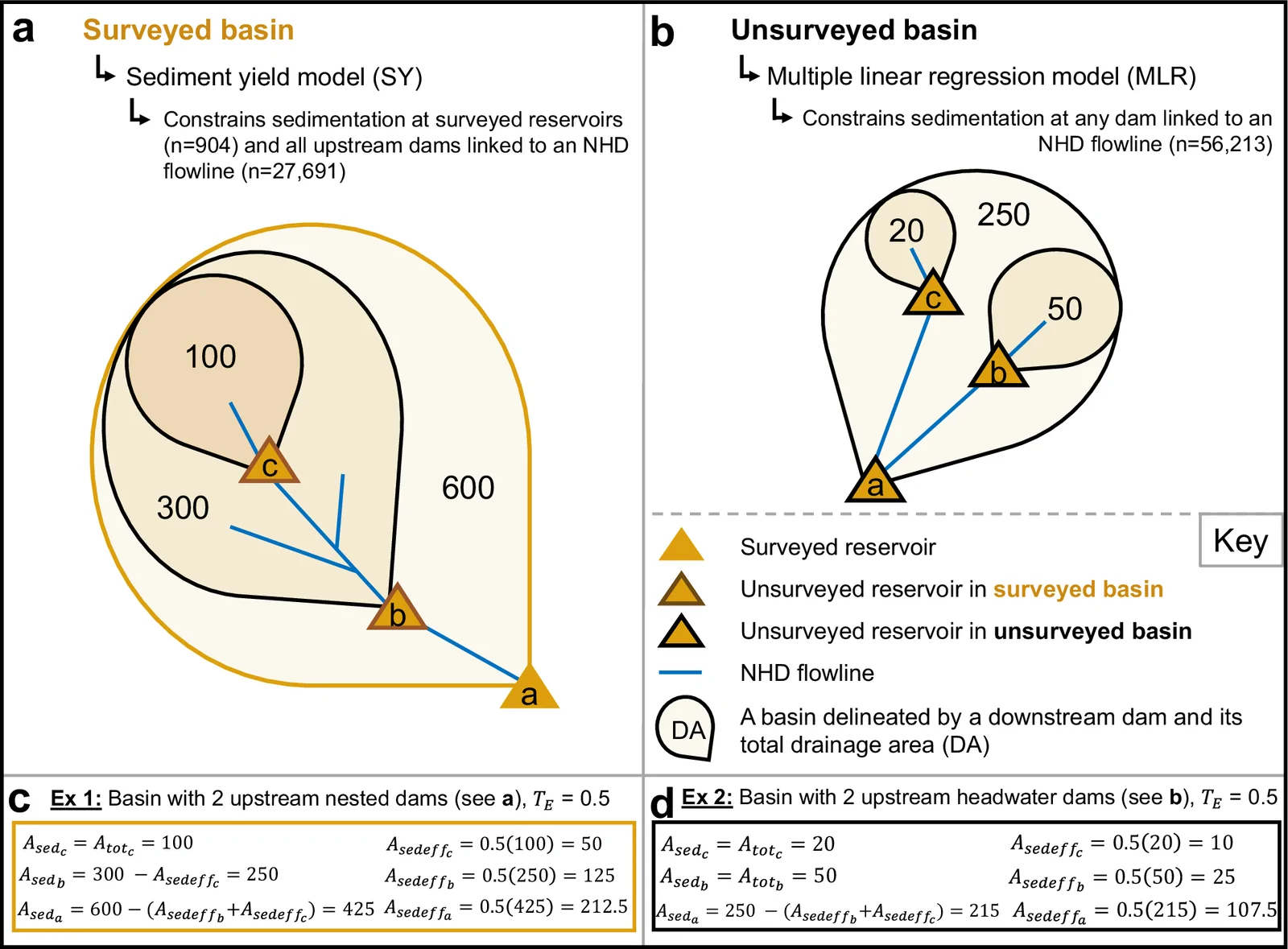
Schematic diagram of RATTES. **a** the sediment yield model for surveyed reservoirs (SY) and **b** the multiple linear regression model for unsurveyed reservoirs (MLR). Example calculations of the effective $({{{\boldsymbol{A}}}}_{{{\boldsymbol{sedeff}}}})$ and sediment-contributing (${{{\boldsymbol{A}}}}_{{{\boldsymbol{sed}}}}$) drainage areas are provided in (**c**, **d**). See “Methods” for equation definitions.

**Fig. 2:**
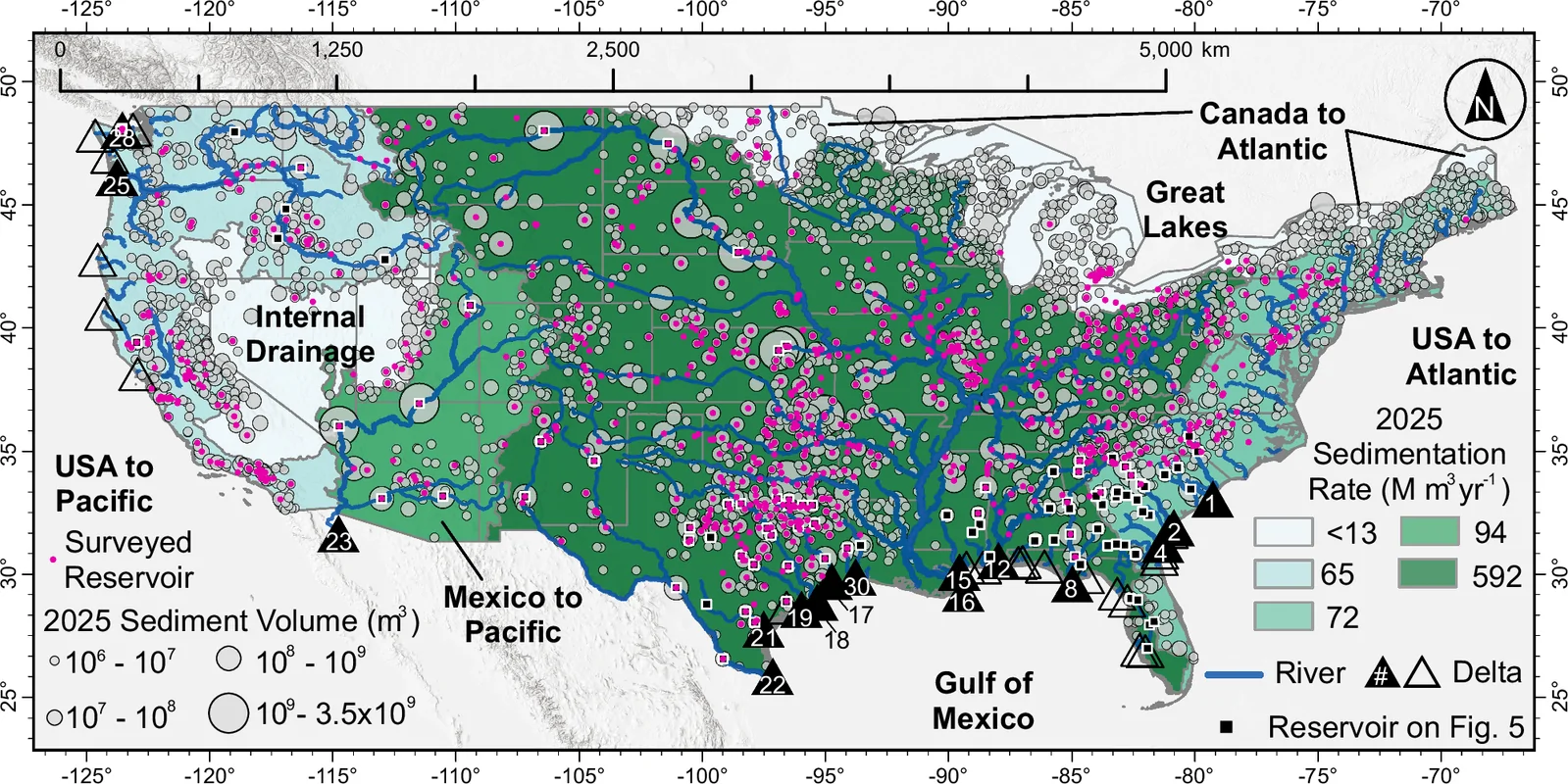
RATTES’ modeled sedimentation in reservoirs across the US, scaled by accumulated reservoir sediment volumes, with minimum value of 1 million m^3^. Small magenta points are surveyed reservoir sites. Map shading corresponds to 2025 total sedimentation rate per year, grouped by ResNet^20^ outlet location (labeled on map). Triangles are delta locations from Edmonds^8^; solid triangles are selected deltas in Fig. 5 and Table 1, with numbers corresponding to delta # in ResNet. Squares indicate up to 5 reservoirs with the largest sediment volumes > 1 M m^3^ above selected deltas and are listed in Supplementary Data 2 (Supplementary Note 2). Hillshade data are from the Esri *Terrain—Multi-Directional Hillshade* layer^88^. State boundaries are from the U.S. Census Bureau TIGER/Line Shapefile (2025)^89^. HUC-8 basin boundaries are originally from the U.S. Geological Survey Watershed Boundary Dataset (WBD)^90^ and are merged into larger basins consistent with ResNet outlet allocations.

RATTES integrates two complementary modeling frameworks: (1) a sediment yield model for surveyed reservoirs (SY) and (2) a statistical sedimentation model for unsurveyed reservoirs employing multiple linear regression (MLR) (Fig. 1 and Supplementary Fig. 1). The SY model numerically solves for a sediment yield rate (m^3^km^−2^yr^−1^) that matches surveyed capacity loss at 904 surveyed reservoirs (Fig. 2) using Minear and Kondolf’s^24^ formulation, originally developed for California but applied here across the US. The MLR model establishes a statistical relationship between environmental predictors to obtain sedimentation rates (m^3^yr^−1^) derived from the surveyed reservoirs. These predictors define RATTES, where *R* is the sedimentation rate between surveys or since reservoir construction (m^3^yr^−1^), ${A}_{{sedMLR}}$ is the arithmetic mean of the sediment-contributing drainage area between surveys or since reservoir construction (km^2^), *T* is the number of years since the previous survey (years), *T*_*E*_ is the linear average dam trap efficiency between surveys^29^ (dimensionless), and *S* is the specific discharge (m^3^km^−2^s^−1^). All variables are transformed via the natural logarithm prior to model fitting. This equation explains 81% of the variance in observed sedimentation rates across surveyed reservoirs (*p*-value: <2.2 × 10^−16^) (Supplementary Table 1).

We parameterize the MLR model to estimate sedimentation rates for 56,213 unsurveyed reservoirs within ResNet using the following equation:

1 $${{\mathrm{ln}}}({R}_{a,t})=0.904{{\mathrm{ln}}}({A}_{{sedML}{R}_{a,t}})-0.148{{\mathrm{ln}}}({T}_{a,t})+1.68{{\mathrm{ln}}}({T}_{E_{a,t-1}})+0.0888{{\mathrm{ln}}}({S_{a}})-1.25$$

where *a* is a given unsurveyed dam and *t* is the timestep in calendar years. For these predictions, *R* and *T* are defined using the number of years between reservoir construction and the current model timestep, while *T*_*E*_ is computed as the linear average trap efficiency from dam completion to the previous timestep. We name our modeling framework and its predictions RATTES, an acronym reflecting the MLR model component that enables sedimentation estimates for more than 98% of reservoirs in the study, including many not previously assessed. Notably, despite investigating dozens of potential predictor variables^37^ (“Methods”), the most important parameters to predict reservoir sedimentation pertain to anthropogenic impacts to the watershed as opposed to climactic or geomorphologic characteristics of basins^38^. Parameters ${A}_{{sedMLR}}$, T, and ${T}_{E}$ all pertain to dam characteristics and contribute 66.8, 7.2, and 7.1% of the total variance in R, respectively, while S, comprising of runoff and total drainage area characteristics, contributes only 0.2%.

### 7.7% of the US reservoir capacity is lost due to sedimentation in 2025

**Fig. 3:**
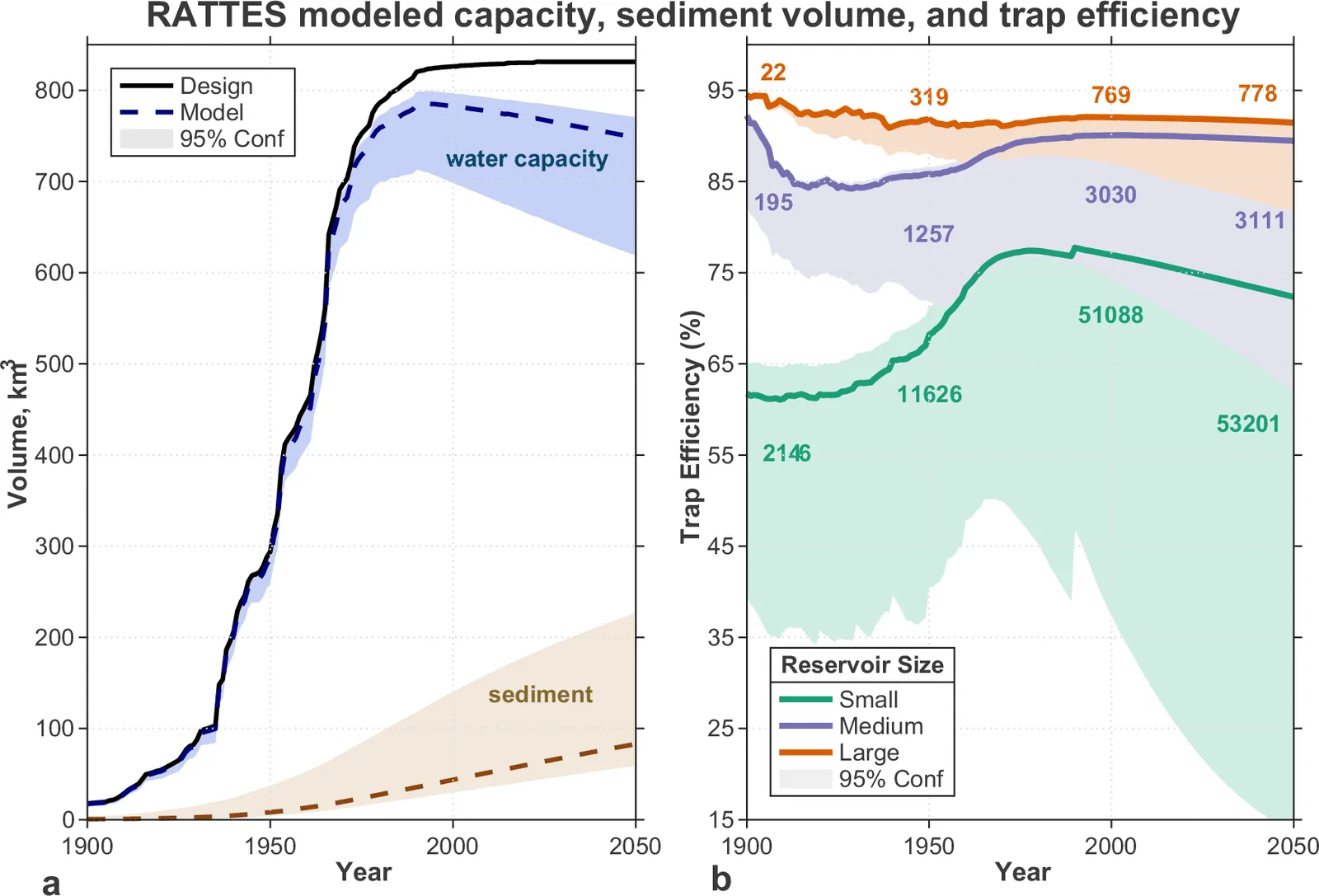
RATTES model results (lines) with 95% confidence intervals (shading) for silt-sized sediment. **a** Available water capacity (blue) and sediment volume (brown) from 1900 to 2050 for 904 surveyed sites and 56,213 unsurveyed reservoirs. **b** Mean trap efficiency for reservoirs. Annotated numbers indicate the number of reservoirs in each category at years 1900, 1950, 2000, and 2050.

The 57,117 reservoirs included in our study represent a design water storage capacity of approximately 832 km^3^. For the 57,090 reservoirs whose dams remain in place through 2025, with a median age of 62 years^20^, we calculate a total capacity loss of 7.7% (Fig. 3a). This loss corresponds to 63.8 km^3^ of sediment, or 61.3 gigatons (Gt), assuming a bulk density of 960 kg m^−3^ ^23,24^ which is 7.2 times the 2010 global annual fluvial sediment load (8.5 Gt)^3^. Similarly, removing the time parameter (T) from Eq. 1 increases the predicted 2025 sediment volume from 63.8 to 65.9 km^3^, consistent with T implicitely accounting for sediment compaction. These and subsequent values assume silt-sized sediment unless explicitly noted otherwise; employing the RATTES model to quantify the cumulative sediment volume assuming clay or sand-sized reservoir sediment yields 60.2 km^3^ of sediment (44.4 Gt using clay density of 737 kg m^−3^)^39^ or 69.6 km^3^ of sediment (103.7 Gt using sand density of 1490 kg m^−3^)^39^, respectively, for year 2025 (Supplementary Figs. 2 and 3).

The US reservoir sedimentation rate in 2025 is modeled at 0.79 km^3^yr^−1^, sufficient to infill nearly 3700 median-sized reservoirs (0.2 M m^3^) each year. By 2050, projected capacity loss at the same 57,090 reservoirs increases to 10.0%, equivalent to 83.0 km^3^ of accumulated sediment (Fig. 3a). The corresponding sedimentation rate in 2050 declines slightly to 0.73 km^3^yr^−1^, slowed because dam trap efficiency declines with water storage capacity loss due to sedimentation, as dictated by Brown^29^ (Fig. 3b).

**Fig. 4:**
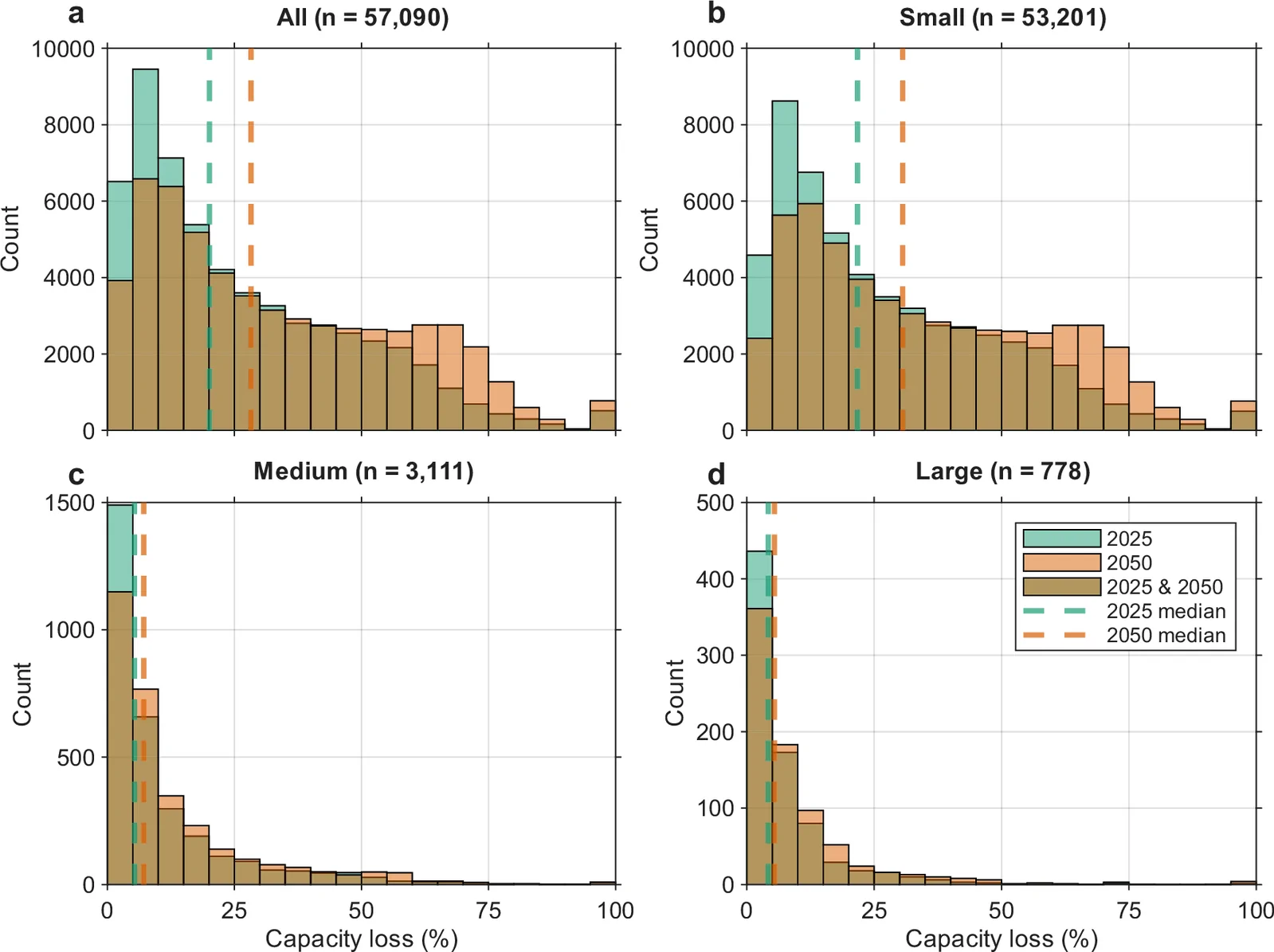
Distribution of total capacity loss among individual reservoirs through 2025 and 2050 by dam size. **a** All dams, **b** small dams (6.2 M m^3^), **c** medium dams (6.2 M m^3^ – 100 M m^3^), and **d** large dams (≥100 M m^3^); n represents the number of dams that remain in place through 2025.

Small and mid-sized reservoirs (< 100 M m^3^ design capacity) have trapped 19.8% (12.6 km^3^) of the total modeled sediment volume by 2025, corresponding with a sedimentation rate of 0.13 km^3^yr^−1^, while large reservoirs trapped sediment at a rate of 0.66 km^3^yr^−1^_._ Thus, small and medium size reservoirs trap a significant amount of sediment that reduces accumulation rates at larger, linked reservoirs downstream, underscoring the need to account for these smaller systems in large-scale assessments^40^ (Fig. 4). The exclusion of sediment trapping at small and mid-sized reservoirs by previous reservoir sedimentation studies^17,18,22,23^ likely overestimated sedimentation at large reservoirs, especially when projecting static sedimentation rates into the future with unchanging trap efficiency. Since RATTES explicitly accounts for both evolving trap efficiency (Fig. 3b) and changing connectivity, distributing sediment throughout the entire dam network, we generate dynamic sedimentation rates and lower capacity losses than other models.

Specifically, RATTES’ total capacity loss estimates are roughly one-third of previous assessments. Perera et al.^17^ estimated 24% total cumulative capacity loss by 2022 and 34% by 2050 for 7469 US reservoirs, assuming constant annual capacity loss rates, no change in trap efficiency, and neglecting the effects of new upstream dams that prolong the design capacity of downstream reservoirs. For California reservoirs, Minear and Kondolf^24^ found that using static trap efficiencies resulted in a 416% increase in modeled sedimentation volumes when compared to their networked sediment yield model incorporating annual changes to trap efficiency. Previous cumulative US sedimentation rate estimates range from 0.49 km^3^yr^−1^ (*n* = 1823)^22^ to 0.84 km^3^yr^−1^ (*n* = 1105)^23^ for mid- to large reservoirs (≥6.2 M m^3^, 5000 acre-feet). Although our study includes 52 times more reservoirs and 7.6 times more initial storage capacity than ref. ^23^ (110 km^3^), our predicted cumulative sedimentation rates fall within a similar range, perhaps because the vast majority of sedimentation is occurring at the 778 large reservoirs with design capacities ≥ 100 M m^3^.

A large fraction of modeled US reservoir sedimentation—66.1%, equivalent to 42.2 km^3^— occurs in reservoirs with repeated survey observations (Fig. 1a). Although surveyed reservoirs represent only 1.6% of the 57,117 reservoirs included in our study, they account for 52.6% of total designed storage capacity and include many of the largest systems, which are more likely to be monitored for capacity loss^25^. By 2050, we estimate that approximately 67.6% (56.1 km^3^) of total US reservoir sedimentation occurs within these surveyed reservoirs, where sediment accumulation has been directly observed over time, increasing confidence in our results. As an example, we demonstrate the close agreement between observed and modeled capacity, sediment volume, and trap efficiency through time for the surveyed site Elephant Butte Reservoir, New Mexico (Supplementary Fig. 4), despite a long history of upstream damming and land use change in the basin^41^.

The median storage loss across all reservoirs is far higher than the national total—20% in 2025 and 28% by 2050 (Fig. 4a)—because cumulative totals are skewed by the available storage volumes at a few extremely large facilities. By 2025, 24,407 reservoirs—43% of current reservoirs—have lost ≥ 25% of their original capacity, which is high enough to potentially impair dam operations. A notable example is Paonia Reservoir in western Colorado, a mid-sized reservoir (0.026 km^3^) in the upper watershed of Muddy Creek that lost roughly 25% of its storage. Sedimentation blocked its intake structure in 2014, prompting costly and ongoing dredging efforts^1^. While Paonia is federally owned, three-quarters of reservoirs that have lost ≥ 25% of their capacity by 2025 are privately-owned outside of local, state, and federal government (*n* = 17,920)^20^. Consequently, the economic burden of sedimentation will fall disproportionately on private dam owners, many of whom may lack the resources or regulatory support available to federal and state agencies.

### Reservoirs store 32.8 km^3^ of sediment above sediment-starved Mississippi River Delta

The Mississippi River basin contains about half of all US reservoir sediment, storing 32.8 of 63.8 km^3^ (Table 1), which is nearly enough sediment to fill Lake Mead (38.1 km^3^, 1936 original capacity), the US’ largest reservoir. The basin with the second-highest sediment storage is the Colorado River basin (8.1 km^3^; 13% of the national total), with 3.5 km^3^ stored in Lake Mead alone, followed by the Columbia River basin (4.2 km^3^; 7%) and the Rio Grande basin (1.8 km^3^; 3%). These estimates reflect sediment storage within US reservoirs only and exclude cross-boundary reservoirs.

**Table 1:** Potential (equivalent) delta aggradation, basin trapping, and terminal dam metrics^85^ computed from RATTES sedimentation results, ResNet^20^, and a global delta area dataset^8^ for selected deltas

Delta Resnet #-Name	Fraction of upstream basin trapped (%)	Dams in upstream basin (#)	Sed volume (M m^3^)	Delta area (km^2^)	Equivalent delta agg., H (m)^a^	Upstream basin sed. rate (M m^3^ yr^−1^)	Equivalent delta agg. rate, h (mm yr^−1^)^a^	Upstream terminal dams (#)	Fraction of sed. in terminal dams (%)
***1-Santee***	65	1838	765	566	0.4	6.4	3.4	245	23
***2-Savannah***	66	641	1484	408	1.1	30.1	22.1	82	1
***4-Altamaha***	40	1150	235	526	0.1	2.5	1.4	420	34
***8-Apalachicola***	78	1163	793	481	0.5	11.0	6.9	3	20
***12-Mobile***	88	1998	1062	253	1.3	15.6	18.5	28	<1
***15-Pearl***	44	482	86	680	0.0	1.3	0.6	260	90
***16-Mississippi***^**b**^	64	28810	32798	53749	0.2	426.2	2.4	3563	4
***17-Trinity***	92	910	976	66	4.5	14.9	68.1	2	<1
***18-Brazos***	78	803	937	681	0.4	13.0	5.8	60	4
***19-Colorado TX***^**c**^	88	565	522	55	2.9	6.5	35.9	2	<1
***21-Nueces***	75	292	143	30	1.4	2.4	23.7	2	35
***22-Rio Grande***	78	407	1809	11806	0.0	20.5	0.5	1	<1
***23- Colorado***^**c**^	93	1045	8123	340	7.2	93.5	82.5	1	<1
***25-Columbia***	90	975	4162	124	10.1	44.9	108.9	45	2
***28-Elwha***^**d**^	[91]	[2]	[19]	[3]	[1.8]	[0.2]	[20.4]	[1]	[16]
***30-Sabine***	65	1838	765	566	0.4	6.4	3.4	245	23

^a^Total equivalent delta aggradation height (H) is the total sediment volume upstream divided by delta area. Annual h rate is the sedimentation rate divided by the delta area. Both assume a 30% delta trapping efficiency.

^b^Includes Atchafalaya/Wax Lake deltas.

^**c**^23 is Colorado River with outlet in Gulf of California, 19 is Colorado River in Texas with outlet in Gulf of Mexico.

^d^All results, except for the Elwha, reference model year 2025. Elwha results in brackets are from model year 2011, prior to dam removals. Supplementary Data 2 extends this table for all 36 deltas (Supplementary Note 2).

**Fig. 5:**
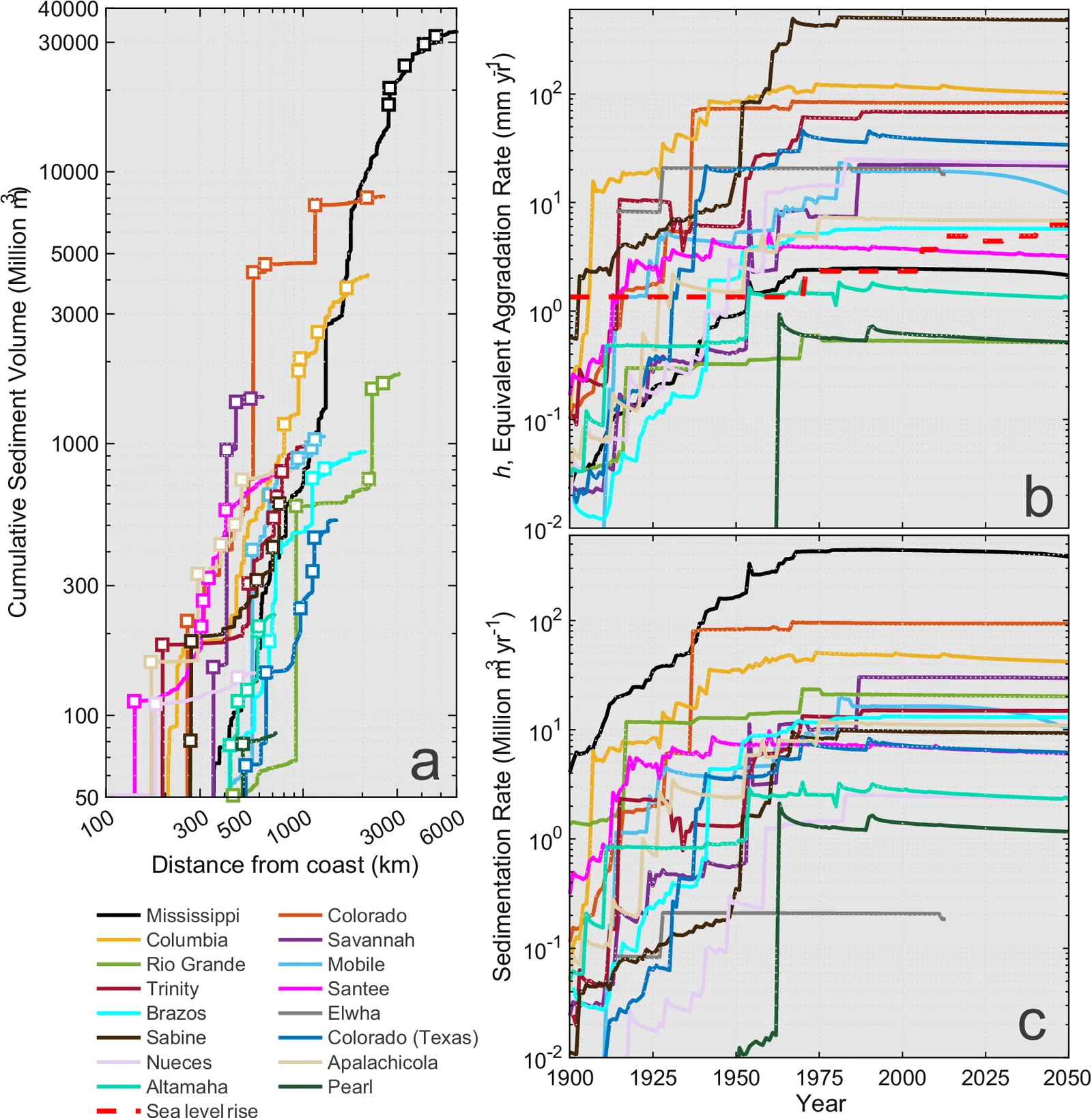
Cumulative reservoir sediment storage upstream from deltas and equivalent annual delta aggradation rate at selected deltas. **a** 2025 Cumulative sediment volume with distance upstream from delta. Square points represent up to 5 of the largest reservoir sediment volumes above each delta, if they exceed 1 M m^3^, and correspond to solid square points on Fig. 2. **b** Equivalent delta aggradation rate (*h*) through time that reservoir sediment could potentially provide to deltas, assuming 30% delta trapping efficiency, compared with eustatic sea-level rise rates. **c** Annual reservoir sedimentation rate in the basin upstream of deltas through time.

Approximately 63% of all US reservoir sedimentation in 2025 occurs within contributing regions to the Gulf of Mexico (Fig. 2). Much of this sedimentation is occurring hundreds to thousands of kilometers inland (Fig. 5a). For example, the five reservoirs that trap the most sediment above the sediment-starved Mississippi Delta^8^ are all situated in the Missouri River basin, more than 2000 km upstream (Fig. 5a and Supplementary Data 2); 25% of all sediment stored in the Mississippi basin is held within these five reservoirs, all designated with the primary purpose of flood risk reduction or hydroelectricity production. Thus, they will likely continue to alter sediment continuity across the continent for decades to come.

### Upstream sediment trapping limits potential delta aggradation

Six of 36 US deltas are located at the outlet of river basins that have trapped more than 1 km^3^ of reservoir sediment by 2025 (Table 1 and Fig. 2), sequestered within 33,876 storage reservoirs. Half of these deltas are located along the Gulf Coast, while the others drain to the South Atlantic, Northern Pacific, and Gulf of California. The surface area of these six deltas span nearly three orders of magnitude, from 124 km^2^ (Columbia) to 53,749 km^2^ (Mississippi)^8^. Despite these differences, all six have more than 60% of their total upstream drainage area impounded by dams, indicating that sediment connectivity is greatly disrupted across their watersheds (Table 1). Further, only four of 36 US deltas are fed by free-flowing rivers, including the Elwha Delta, whose upstream dams were removed between 2011 and 2014 (Fig. 5b); the remaining 32 deltas have upstream dams that constrain sediment supply.

To explore the impact of upstream damming on coastal delta sedimentation, we estimate the total equivalent length-scale of delta aggradation (*H*). These values range from <0.01 m to 31.2 m for deltas with upstream dams (Table 1 and Supplementary Data 2). *H* depends on the total modeled volume of sediment upstream, a commonly used dataset of total delta surface area^8^, the assumption that all sediment currently retained in reservoirs would otherwise have been delivered to the coast, and that 30% of this reservoir sediment would be retained in subaerial delta plains^7^ (“Methods”). Realistically, some fraction of the sediment stored in reservoirs would be trapped by other depositional environments such as upstream floodplains or natural lakes. Also, some delta surface areas are very small, and these systems experience higher bypass, resulting in unrealistically high aggradation rates for a few deltas. Our method does not have an aggradation limit, whereas in a natural delta, sustained floodplain aggradation subsequently reduces flood inundation and limits subsequent aggradation. Nonetheless, these potential aggradation heights provide a useful visualization of the magnitude of sediment sequestration upstream and the potential scale of sediment deprivation at individual deltas.

The annual equivalent delta aggradation rates (*h*) range from <0.02 mm yr^−1^ to 486 mm yr^−1^ for 2025 (Table 1 and Supplementary Data 2). The Mississippi Delta lies toward the lower end of this range, with an estimated 0.2 m *H* and 2.4 mm yr^−1^ *h* in 2025. This rate is similar to its long-term, time-averaged rate of sediment accumulation determined from radiocarbon dating (2.6 mm yr^−1^, 11−3.5 kyr BP)^42^. These rates reflect the inclusion of the delta’s entire historical area, rather than only the currently active delta complexes receiving flow and sediment (Atchafalaya-Wax Lake: 2800 km^2^ and Plaquemines-Balize: 9930 km^2^)^43,44^. Reducing the delta area to the presently active lobes (12,730 km^2^) yields equivalent depth and aggradation rates roughly four times higher (0.8 m and 10.0 mm yr^−1^ in 2025). Note that these rates do not account for the modern levee network that prevents overbank flooding and natural sediment dispersal across the delta plain. Nevertheless, without major Missouri River basin dams and levee systems, sediment fluxes to both active delta complexes would be substantially greater, enough for aggradation rates (10.0 mm yr^−1^) to potentially keep pace with present-day rates of both eustatic (4.4 mm yr^−1^)^43^ and relative SLR (7.3 mm yr^−1^)^45^.

All deltas exhibit increasing *h* through the early-to-mid 20th century (Fig. 5b), coincident with rapid dam construction, followed by a pronounced slowdown during the 1970s−1980s as dam building waned. Beyond 2000, *h* typically declines gradually toward 2050, reflecting reduced reservoir trap efficiency as dams age and pass a slightly larger fraction of sediment downstream (Fig. 3b). Currently, *h* exceeds eustatic SLR at almost half of US deltas (17/36) (Fig. 5b, Supplementary Data 2, and Supplementary Table 2). However, relative SLR is known to be especially rapid in the Gulf of Mexico^46,47^. If there were no inland dams, sedimentation across the entire Mississippi and Rio Grande deltas would still likely not keep pace with eustatic SLR (Fig. 5b), highlighting the enhanced vulnerability of Gulf Coast deltas to ongoing global SLR, which is amplified by local land subsidence^45^. For instance, *h* for the Rio Grande delta is only 0.5 mm yr^−1^, whereas its current relative SLR rate is nearly 14 times higher (6.8 mm yr^−1^)^45^.

Although some studies have reported increased coastal sedimentation or little change in sand transport despite widespread dam construction^26,28^, historical observations indicate that sediment delivery to US coasts has generally declined, partly because of dams^2,3,6,48,49^. RATTES does not directly model coastal sediment flux because it estimates sediment yield only within the dammed portion of each basin (Fig. 1). To provide a first-order comparison, we extrapolate the modeled yield from the dammed area across the full basin upstream of each delta (Supplementary Note 3 and Supplementary Data 3). These whole-basin estimates commonly exceed historical coastal fluxes because they assume spatially uniform sediment yield, whereas sediment yield generally decreases with increasing drainage area^48^.

Nevertheless, the extrapolated estimates are generally within an order of magnitude of historic and modern observations. For example, although the Mississippi-Atchafalaya has maintained relatively stable sand (bedload) transport in recent decades^26^, suspended sediment flux has declined from approximately 378 M m^3^yr^−1^ around 1900 to 159 M m^3^yr^−1^ today^49^, a 58% reduction. By comparison, the extrapolated 2025 whole-basin sedimentation rate is 665 M m^3^yr^−1^. Its higher value is consistent with anthropogenic enhancement of watershed erosion occurring alongside reduced sediment delivery to the coast,^3,28^. A remarkable example of sediment flux decline to the coast is that of the Colorado River, which transported an estimated 184 M m^3^yr^−1^ before dam construction but now delivers almost no sediment to the Gulf of California^50^. RATTES estimates a whole-basin sediment yield of 100 M m^3^yr^−1^ today, roughly half of the pre-dam coastal flux (Supplementary Data 3). This difference likely reflects both spatial variation in sediment yield and reduced sediment transport caused by drought, flow regulation, and flood control^50^. Together, these comparisons suggest that substantial sediment production persists within dammed basins (Fig. 5c), but dams and flow regulation limit its delivery to the coast, reducing the capacity of downstream deltas to aggrade and keep pace with SLR (Fig. 5b).

### Terminal reservoirs offer opportunities to reinstate sediment connectivity

Although approximately 79% of the 2025 modeled US reservoir sediment volume resides more than 500 km inland from the coast, about 7% (4.5 km^3^) is stored in terminal reservoirs impounded by the downstream-most dam along a river. Reinstating sediment passage at these dams would reconnect sediment transport to coastal and deltaic systems.

Along the Gulf Coast, the Brazos River basin has 60 terminal dams storing ~36.9 M m^3^ of sediment (Table 1), and the Trinity and Apalachicola basins hold tens to hundreds of millions of cubic meters only a few hundred kilometers from the shoreline (Fig. 5a). These deposits represent potentially viable, and relatively proximal, sources of sediment for delta nourishment and coastal restoration. In contrast, the Mississippi River Delta has 3563 upstream terminal dams located deep into the continental interior^20^ (Table 1). These dams store ~1.1 km^3^ of sediment—less than 4% of the total reservoir sediment upstream of the Mississippi Delta—yet nearly enough to fill Lake Maurepas, Louisiana, twice (0.65 km^3^)^51^. Reconnecting sediment transport through or around a small number of terminal reservoirs with high sedimentation rates would provide greater potential benefits than efforts to reconnect sediment continuity in basins with sediment distributed across numerous terminal dams.

Dam removals along the Elwha and Klamath Rivers, located on the western coast of the US, provide examples of terminal dam removals that reinstate ecosystem services and sediment transport^13^. Each basin had a single terminal dam on the mainstem; removing it, along with additional upstream structures, increased the impact of these projects. Our model indicates cumulative sedimentation rates behind removed dams of ~0.2 M m^3^yr^−1^ for Elwha River reservoirs and ~0.1 M m^3^yr^−1^ for Klamath River reservoirs, sediment that is now mobile. Within 5 years of the Elwha dam removals, 65% of all impounded sediment (19.3 of 30 Mt) was eroded, 26% of which was deposited in the Elwha Delta^52^. The delta area increased by 0.3 km^2^, much of which was colonized by vegetation^13^.

However, dam removals are rare because reservoirs provide critical water storage, flood control, irrigation, and hydropower benefits. At critical dams, sustainable reservoir management techniques can reduce sedimentation, increase water storage resilience, and restore downstream sediment delivery^53^. Efforts to reconnect sediment transport networks recognize that sediment, like water, is a resource requiring sustainable management^15,54^. Targeting sustainable management at terminal reservoirs offers the greatest potential to increase coastal sediment supply without impacting downstream reservoirs. Sediment bypassing, flushing stored sediment, and sluicing entrained sediment (i.e., passing a density current through dam gates) can slow sedimentation rates and even recover lost storage.

Tuttle Creek Reservoir near Manhattan, Kansas—the US reservoir with the largest stored sediment volume (3.0 km^3^)— is currently testing the first water-injection dredging experiment at an inland reservoir. The technique creates gravity-driven density currents to release sediment loads through the dam into the downstream river ecosystem previously experiencing channel degradation and sediment scarcity^55–57^. Tuttle Creek has only one small hydroelectric lock dam located downstream on the Missouri River. Because sediment can more easily pass through navigable lock dams due to their operational style (e.g., on the Mississippi River), sediment released from Tuttle Creek may have the potential to reach the coast. This technique may not be beneficial everywhere; silt-laden releases negatively impact natural gravel-bed streams that support species like salmon. Reservoirs on naturally turbid rivers remain the best candidates for sediment releases from reservoir beds, where species like the endangered humpback chub can benefit from restored turbidity^58^.

### Reservoirs store sediment resources worth billions of dollars

Dams efficiently trap coarse sediment^59^, redistributing sand and gravel into thousands of reservoirs. These materials are critical for infrastructure, housing, and shoreline protection and are becoming increasingly scarce^60,61^. In 2024, the US produced ~890 million tons of construction sand and gravel valued at ~$12 billion^62^. Using this estimate, if only 15% of the modeled reservoir sedimentation in 2025 consists of sand and gravel, its approximate value is $124 billion.

Reservoir sediment represents an underutilized resource for river and coastal restoration. Beach nourishment projects often utilize offshore sediment^63,64^ despite evidence that offshore extraction can exacerbate coastal erosion and degrade benthic habitats^65^. Similarly, sediment augmentation projects for degraded rivers commonly overlook opportunities to salvage reservoir sediment or repurpose removed river sediment^59^. With the global demand of sand rising to over 500 billion tons by 2100^61,66^, reservoir sediments offer a valuable source for river and coastal restoration^15^ that could reduce reliance on offshore sand extraction.

### Broader implications of RATTES

Here, we present a spatially and temporally explicit reconstruction of reservoir sediment storage across the contiguous US, spanning more than 57,000 reservoirs over more than three centuries. We estimate that inland dams have collectively stranded 63.8 km^3^ of sediment by 2025—more than seven times the annual global sediment load to the oceans. Without inland dams, the potential sediment delivery at half of US deltas could keep pace with present-day sea level rise. Further, only four US deltas are located downstream from free-flowing rivers; one of these includes the Elwha River delta, which is actively prograding following dam removal.

Many studies of delta degradation attribute sediment starvation broadly to inland damming but treat reservoirs as static sediment sinks. By resolving where, when, and in which reservoirs sediment is stored as a linked network, our work reframes inland reservoirs as potential sediment resources rather than permanent losses. Our results identify opportunities to mitigate sediment trapping—particularly at terminal reservoirs—to augment storage capacity and enhance sediment delivery to downstream river and delta systems.

Although uncertainty at individual reservoirs can be substantial, RATTES provides a first-order assessment for identifying reservoirs and river basins most vulnerable to sedimentation impacts, helping prioritize bathymetric surveys and targeted data collection to assess potential infrastructure risks. Further, the RATTES dataset establishes a foundation for future studies to quantify: (1) how reservoir sedimentation influences carbon storage and biogeochemical cycling^22,67^, (2) the long-term sustainability of US water storage infrastructure, and (3) the economic tradeoffs among dredging, sediment bypass, and dam removal strategies^68^. By quantifying sediment storage across reservoir networks, RATTES helps clarify how inland dam management may shape future sediment delivery to vulnerable coastal deltas.

## Methods

### Datasets

#### ResNet

ResNet joins 57,307 US dams and 145 major river mouths to NHDPlusV2 flowlines^21^, integrating attributes from the NHD, the National Inventory of Dams^69^, global dam datasets^70–73^, and additional derived parameters, like coastal outlet and streamwise distance to the coast^20^. The resulting connected dam network enables calculation of the upstream sediment-contributing drainage area (${{A}_{{sed}}}_{a}$) upstream of any given dam a on an annual basis, a key input to the sedimentation models that updates as dams are built or removed.

Within ResNet, 9545 reservoirs lack a reported dam completion year, which is needed to estimate reservoir sedimentation using the models developed in this study; although this is a significant number of reservoirs, they contain less than 1% of total US water storage capacity. For these dams, we set the dam completion year to the 90th percentile of reported dam completion dates, 1990; we chose the 90th percentile because we did not want to overestimate sedimentation at these reservoirs and thus overestimate the number of reservoirs experiencing high levels of capacity loss. As a result, we likely underpredict sedimentation at these reservoirs, as it is more probable that they were constructed earlier (median completion year of dams remaining in 2025: 1963).

An exception is made for Soo Locks, which the U.S. Army Corps of Engineers reports as having 342 km^3^ of reservoir capacity, including substantial storage from Lake Superior. Most of the upstream watershed lies within the Great Lakes, which have been trapping sediment well before the emplacement of Soo Locks. We therefore set the year of dam completion for Soo Locks, Michigan, to 1700, which corresponds to the model start; emplacing Soo Locks at the start of the model to “dam” Lake Superior more realistically represents actual sediment trapping.

We identify 190 reservoirs without permanent storage, including Soo Locks, which are typically navigable lock dam structures that lack the capability to permanently impound water, hence passing up to 100% of incoming sediment through the dam^74^. We visually inspect these structures using satellite imagery and confirm that they do not appear to have operable dam gates capable of impounding a pool of water. Although we do not predict sedimentation at reservoirs without permanent storage, we retain them in the dam network and account for them in the ${A}_{{sed}}$ computation. Thus, we predict sedimentation at a total of 57,117 reservoirs (57,307 dams in ResNet minus 190 navigable dams). We also incorporate eight major Canadian reservoirs into our ResNet run to accurately represent the ${A}_{{sed}}$ on the Columbia River system (Mica, Revelstoke, Keenleyside, Corra Linn, Brilliant, Seven Mile, Waneta, Duncan; Supplementary Data 1), but we do not report sedimentation at these reservoirs. These same eight Canadian reservoirs were incorporated in the sediment-contributing drainage area example detailed in the paper describing ResNet^20^.

#### Sedimentation survey data

We compile repeat reservoir survey data for 904 US reservoirs (Supplementary Data 1, Supplementary Note 1, and Supplementary Fig. 1). We exclude survey data where (1) reported water capacity increased through time, likely caused by poor original survey data, changing survey methodology, or dam raises, and (2) the interval between surveys was less than 10 years to allow sufficient time for discernable sediment deposition. We typically use the first and last available survey data, that meet the above two conditions, to maximize available time for sediment deposition; exceptions occur when the original data are incomplete or there was a dam raise.

We calculate sediment volume (*V*_*sed*_) (m^3^) as the change in water storage capacity between the two selected surveys, assuming all capacity loss is driven by sedimentation (like past studies^17,18,22–24,75^). The link between reservoir sedimentation and capacity loss is validated in many bathymetric reports, which document an increase in reservoir bottom elevation through time due to sediment deposition and, less commonly, through the collection of sediment cores or geophysical surveying^76,77^ (Supplementary Note 4). Realistically, a portion of the capacity change reflects compaction of previously deposited sediments. Since the median interval between surveys is 35 years, capacity loss is mainly driven by sedimentation with loss rates reducing through time as sediment compacts. Compaction is therefore implicitly incorporated into our models through the survey-derived capacity changes. Explicitly representing compaction would require reservoir-specific information on operations and sediment properties^39,78^ that is not available at the national scale.

In addition to the 904 surveyed reservoirs, we obtain survey data for another 20 reservoirs with navigable locks and thus no permanent storage. We do not predict future sedimentation (past the second survey) and the statistical model is not trained on data at these 20 sites because they are operated differently and likely dredged. However, we incorporate surveyed sedimentation at these reservoirs into the sediment yield model because it provides the best available data to calculate ${A}_{{sed}}$.

### Components of RATTES

#### Sediment yield model for surveyed reservoirs

The sediment yield (SY) model numerically solves for a sediment yield rate (m^3^km^−2^yr^−1^) using the surveyed reservoir water storage capacities, accumulation of sediment at all upstream dams (surveyed and unsurveyed) after dam emplacement, and time-varying dam trapping efficiency (Fig. 1a and Supplementary Fig. 1). The model projects sedimentation at any of the surveyed reservoirs into the future, assuming that sediment yield rates (Eq. (2)) are constant with time. The model follows the method of Minear and Kondolf^24^, but by implementing a numerical solution, we do not have to follow their assumption of a linear change in trap efficiency between surveys, and allow the trap efficiency to update at every time step. The model steps through time, accumulating sediment at upstream reservoirs and adjusting the sediment-contributing drainage area and trap efficiency for all dams at every timestep (*dt* = 1 year) (Eqs. (3)–(5); with these changing parameters, the model solves for the sediment yield rate that can produce the surveyed reservoir capacity at the last survey within 0.01%. For unsurveyed reservoirs located upstream from surveyed sites, the model applies the sediment yield at the closest downstream, surveyed reservoir. The SY model component enables the incorporation of reservoir survey data from differing time periods on multiple reservoir subbasins, including nested reservoir subbasins (Fig. 1a), to constrain sediment yield rates within larger upstream basins.

A volumetric sediment yield rate at reservoir *a*, ${Y}_{a}$ (m^3^km^−2^yr^−1^), is equivalent to the calculated volume of sediment divided by the time between surveys and time-weighted effective sediment-contributing drainage area (${A}_{{sed}{{eff}}_{a,\bar{t}}}$)^24^, such that:

2 $${Y}_{a}=\frac{{V}_{{se}{d}_{a}}}{\left({t}_{a,{{survey}}_{2}}-{t}_{a,{{survey}}_{1}}\right){A}_{{sed}{{eff}}_{a,\bar{t}}}}$$

where ${V}_{{se}{d}_{a}}$ is the sediment volume in m^3^, and ${t}_{{survey}}$ represent the first and last survey years. ${A}_{{sed}{{eff}}_{a,\bar{t}}}$ accounts for trapping efficiency at reservoir *a* through time (following Brown^29^) and the emplacement and trapping efficiency of upstream dams and reservoirs (*b*, *c*, *d*, …), which affect the time-dependent sediment-contributing drainage area^24^:

3 $${A}_{{{sed}}_{a,t}}={A}_{{{tot}}_{a}}-\left[{T}_{{E}_{b,t}}\left({A}_{{{sed}}_{b}}\right)+{T}_{{E}_{c,t}}\left({A}_{{{sed}}_{c}}\right)+{T}_{{E}_{d,t}}\left({A}_{{{sed}}_{d}}\right)+\ldots \right]$$

4 $${A}_{{{sedeff}}_{a,t}}=\left({A}_{{{sed}}_{a,t}}\right)\left({T}_{{E}_{a,t}}\right)$$

*A*_*tot*_ is the total basin drainage area and is static through time. For nested reservoirs, *A*_*sed*_ must be calculated moving from upstream to downstream basins; at headwater reservoirs with no additional upstream dams, *A*_*sed*_ is equivalent to *A*_*tot*_. We apply a trap efficiency, *T*_*E*_, following Brown^29^ for reservoir *a* at time *t*:

5 $${T}_{{E}_{a,t}}=1-\frac{1}{1+0.0021\kappa \left(\frac{{V}_{{ca}{p}_{a,t}}}{{A}_{{{tot}}_{a}}}\right)}$$

where κ is a coefficient representing sediment particle size retained in the reservoir and ${{V}_{{cap}}}_{a,t}$ is reservoir a’s water capacity at timestep *t* (calendar year). For our models, we assume silt as the dominant grain size mode retained in reservoirs (κ=0.1)^35^. We run two additional scenarios to determine upper and lower bounds for trap efficiency by assuming all study reservoirs trap sand (κ=1) and clay (κ=0.046).

At reservoirs with survey data, the sedimentation model treats the first surveyed reservoir capacity as a known data point and iteratively solves for a volumetric sediment yield rate that produces a reservoir capacity within 0.01% of the reported capacity for the second survey $({{V}_{cap}}_{a,t},{{\rm{where}}}\,{t}={t}_{a,{{survey}}_{2}}).$

Overall, 28,623 reservoirs are included in the SY model, including the 904 surveyed reservoirs with permanent storage, 20 surveyed reservoirs without permanent storage, and 27,699 unsurveyed reservoirs (Fig. 1a). Once we solve for a sediment yield rate, we project future sedimentation at the permanent storage surveyed sites beyond the last survey year $({t}_{a,{{survey}}_{2}}).$ If the first survey year $({t}_{a,{{survey}}_{1}})$ post-dates the dam completion year, we use the sediment yield rate to back-project sedimentation from the first survey date to the dam’s year of completion.

#### Multiple linear regression model for unsurveyed reservoirs

We develop a statistical sedimentation model to predict sedimentation at dams located both upstream of the 904 surveyed site basins and in completely unsurveyed basins (*n* = 56,213) (Fig. 1b). It is a MLR model that incorporates time-varying sediment-contributing drainage area, trap efficiency, and watershed metrics derived from ResNet^20^. It is calibrated using the repeat survey data from 904 US reservoirs (Supplementary Fig. 1), with the natural logarithm of the observed sedimentation rate $({{\mathrm{ln}}}\left(R\right))$ as the response variable to allow for variability across reservoirs spanning a wide range of sizes, ages, and positions within the dam network. Because the model is trained on past survey data, it assumes that sedimentation trends in the future will mimic the past.

We compute the sediment-contributing drainage area for all 56,213 unsurveyed reservoirs, tracking upstream dam emplacement through time. For these computations, we use the static capacities reported by ResNet^20^. Unlike the SY model, which updates the sediment-contributing drainage areas each year using modeled capacities, the MLR model implements Eqs. (3)–(5) using ResNet design capacities, trap efficiencies, and completion year data^20^ to track the emplacement of reservoirs through time, where *a* is a given unsurveyed reservoir. We parameterize this calculation of sediment-contributing drainage area, ${A}_{{sedMLR}}$, to train the model because it allows for a consistent calculation of the sediment-contributing drainage area that can also be implemented at unsurveyed basins for model predictions. The ${T}_{E}$ at dam a itself is parameterized as the linear average ${T}_{E}$ between first and last surveys. From the ResNet output^20^, we also compute the specific discharge, *S*, as mean annual modeled inflow divided by *A*_*tot*_ to represent basin runoff yield.

Prior to building the MLR model, we pre-process the dam watershed parameter data derived from ResNet^20^ and other sources^79,80^. We filter and transform the data to ensure the statistical requirements for MLR are met, including linearity, homoscedasticity, normality, and no multicollinearity. We remove *A*_*tot*_ from the parameter dataset due to its strong collinearity with ${A}_{sedMLR},$ the latter of which explains a greater proportion of variance in observed ${{\mathrm{ln}}}(R).$ We normalize both predictor and response variables to identify and remove outliers by calculating z-scores, defined as the number of standard deviations a data point deviates from the mean of a dataset. We define outliers as observations with an absolute z-score greater than five. Finally, we apply a natural logarithmic transformation to all parameters to account for the wide range of reservoir and basin sizes. More details on the dam watershed parameters explored and processing steps are provided in Eckland, Chapter IV^37^.

We then build the MLR model using the “regsubsets” function in the *leaps* package^81^ in RStudio, which selects the best combination of independent variables to predict ${{\mathrm{ln}}}(R)$ (m^3^yr^−1^). Model selection is based on the Bayesian Information Criterion, which balances goodness of fit with model complexity by penalizing overly parameterized models. The only variable we forced into the model is the time between reservoir surveys (T), which reflects the degree of sediment compaction as reservoirs age. For predictions, ${T}_{t}$ at time *t* is defined as the number of years since the year with the first available capacity data, *yrp*; *T* is an essential parameter because model predictions extend far beyond the median survey interval (35 years).

The full selected model contains 11 parameters; however, four of these parameters explain 82% of the variance in *R*, whereas the remaining seven together explain only an additional 5%. Thus, we reduce the model to the four-parameter formulation presented in Eq. (1). Cross-validating the model by randomly removing 30% of the surveyed site data and training on the remaining 70% produced nearly identical explanatory power (*R*^2^ = 0.809) (Supplementary Table 1). Supplementary Fig. 5 contains model performance visualizations for the MLR model and its training dataset.

The sedimentation rate (R, m^3^yr^−1^) at calendar year t is obtained by exponentiating the regression prediction:

6 $${R}_{a,t}={e}^{{{\mathrm{ln}}}({R_{a,t}})\pm \in }$$

Reservoir storage capacity, C (m^3^), at time, t, is updated in reference to ${C}_{{yrp}},$ which is the first reported capacity data available, such that:

7 $${C}_{a,t}={C}_{a,{yrp}}-{T}_{a,t}({R}_{a,t}){{\rm{\pm }}}\in$$

This method to update ${C}_{t}$ is used as opposed to referencing the previous year’s capacity, ${C}_{t-1}$, and an annual ${R}_{t}$ to maintain consistency with how the MLR model is developed. The model is calibrated on data where only two capacity values were known from at least 10 years apart. Thus, R at surveyed sites represents an average over all years between surveys. Finally, ${T}_{{E}_{a,t}}$ is updated according to Brown^29^ (Eq. (5)). Like all previous years, future predictions (through 2050) utilize parameters that change with respect to the dam network (${A}_{{sedML}{R}_{a,t}},{T}_{a,t},$ and ${T}_{{E}_{a,t-1}}$) as trap efficiency wanes and sedimentation progresses, while ${{S}}_{a}$ remains static (Eq. (1)).

Similar to the SY methods, if the year of the first reported capacity data (*yrp*) post-dates the year of dam completion, we use the sediment yield rates to back-project reservoir sedimentation to the year of dam completion. In the MLR model component, sediment yield is not static with time. Here we use the mean sediment yield rate between *yrp* and the last year reporting sedimentation—either year 2050 (model end), the year prior to dam removal, or the last year prior to complete sediment infilling and capacity loss at a reservoir.

### Uncertainty estimation

We quantify the absolute uncertainty for the annualized capacity and sedimentation predictions using 95% confidence intervals (CI), derived from the MLR model’s residual standard error (Supplementary Table 1). Error for the upper bound of the CI is greater than that for the lower bound because the CI is computed for the natural logarithm of the sedimentation rate, $({{\mathrm{ln}}}(R)).$ Uncertainty is not computed for the SY model, since it would originate from any remaining capacity and drainage area errors from ResNet, which was already rigorously QC-ed^20^, or from the Brown^29^ trap efficiency equation itself (Eq. (5)).

### Delta metrics

There are 36 major coastal deltas in the contiguous US with reported subaerial surface areas included in the global delta dataset of Edmonds^8^. This dataset provides spatially consistent delineations of deltas derived using a uniform methodology.

For each delta at model year 2025, we cumulate the total sediment volume stored in upstream reservoirs (km^3^) and normalize this volume by delta area (km^2^). We then multiply this depth by 0.3, assuming that deltas retain 30% of the sediment they receive^7^. The resulting aggradation height (*H*, m) represents the total potential sediment aggradation that deltas could have accumulated if upstream reservoirs did not exist and all sediment currently stored in reservoirs reached deltas. This assumption likely underestimates true aggradation because it does not account for sediment that bypasses dams and reaches deltas.

We also calculate potential annual delta aggradation rates by summing upstream sedimentation rates for each delta annually and applying the same retention and area-normalization steps described above. This yields an annual equivalent aggradation rate (*h*, mm yr^−1^), representing the thickness of sediment that could potentially be retained by deltas each year if sediment was not stored upstream in reservoirs. These aggradation rates allow direct comparison with reconstructed and projected rates of SLR (for simplicity, we use global SLR rates from Fox-Kemper^82^) (Supplementary Table 2).

Finally, we use ResNet’s terminal dam identifier and reported streamwise distance to the coast (“pathx”) to quantify the cumulative distance from the coast and to estimate the volume and proportion of sediment stored in terminal dams. The count of terminal dams is taken from ResNet and is treated as static (i.e., not updated to reflect dam removals). The sediment volumes stored at terminal dams are derived from the model and therefore reflect the removal of a terminal dam through time.

### Sediment yield and multiple linear regression model comparison

The RATTES model reports results from the SY model at surveyed reservoirs (*n* = 904) and the MLR model at unsurveyed reservoirs (*n* = 56,213). However, the SY model also generates sedimentation predictions at the 27,691 unsurveyed reservoirs located upstream from the 904 surveyed reservoirs with permanent storage (Fig. 1a). We report the MLR model results for all 56,213 unsurveyed reservoirs to apply a consistent methodology across the US. To compare sedimentation predictions made by the SY versus the MLR model, the SY model predicts 7.9 km^3^ of sedimentation in year 2025 while the MLR model predicts 8.6 km^3^ of sedimentation (Supplementary Note 5). Supplementary Figs. 6−10 compare SY and MLR capacity and sediment volume predictions through time (1900–2050) and for select years (2025, 2050).

## Supplementary information

Supplementary Information

Description of Additional Supplementary Files

Supplementary Data 1

Supplementary Data 2

Supplementary Data 3

Transparent Peer Review file

## Data Availability

We obtained reservoir survey data from various sources, complied in Supplementary Data 1, which we incorporated into ResNet v.1 (https://doi.org/10.5281/zenodo.15644268)^83^. Sedimentation and capacity timeseries results, including uncertainty bounds, for reservoirs in RATTES for years 1700 to 2050 are available at Zenodo (https://doi.org/10.5281/zenodo.20789549)^84^. Timeseries results for reservoir/dam impacts above coastal deltas for years 1700 to 2050 are also available at Zenodo (https://doi.org/10.5281/zenodo.21692937)^85^.

## Code availability

The tools used to perform this analysis included: ResNet^20^, run with Python (version 3.9.18) and arcpy module (ArcGIS version 3.2). The SY model in Matlab (version 2025b), MLR model in RStudio (version 2025.05.1 + 513), and model combination and final analysis in Matlab are openly available on GitHub (https://github.com/abbyeckland/rattes-naturecomms.git)^86^ and Zenodo (https://doi.org/10.5281/zenodo.21690856)^87^.
