# Supplementary material for: Reservoir sedimentation diminishes water storage and coastal delta resiliency
Source: Nat Commun. 2026 Aug 25;17:10164. doi: 10.1038/s41467-026-76986-3 (PMC13601615; doi:10.1038/s41467-026-76986-3)
Supplement: Supplementary file 1 [file 41467_2026_76986_MOESM1_ESM.pdf]

Supplementary Materials for:

Reservoir sedimentation diminishes water storage and coastal delta resiliency

Abigail C. Eckland[1,2*], Melissa A. Foster[3*], Aaron A. Hurst[4], Mussie T. Beyene[3], Irina Overeem[2]

[1] University of New Orleans, Department of Earth & Environmental Sciences, New Orleans, Louisiana, USA

[2] University of Colorado at Boulder, Department of Geological Sciences and INSTAAR, Boulder, Colorado, USA

[3] Bureau of Reclamation, Technical Service Center, Denver, Colorado, USA

[4] Utah State University, Department of Civil and Environmental Engineering and Utah Water Research Laboratory, Logan, Utah, USA

*Corresponding Authors:

Abigail Eckland, aeckland@lsuneworleans.edu; Melissa Foster, mfoster@usbr.gov

Contents of this file:

Supplementary Notes 1-5;

Supplementary Methods 1;

Supplementary Figures 1-11;

Supplementary Tables 1-3.

Supplementary Note 1. Site data used to regenerate ResNet.

Supplementary Data 1 includes an xlsx file of surveyed reservoir data, updated capacity data, data sources, and any other data used to regenerate the ResNet dataset[1,2].

Supplementary Note 2. Delta data.

Supplementary Data 2 includes a csv file of sedimentation above US deltas, expanded from the selected deltas in Table 1 of the paper to all 36 US deltas. This table also includes the ResNet Short ID numbers for the reservoirs with the 5 largest sediment volumes upstream from each delta.

Supplementary Note 3. Sediment fluxes to select deltas through time: Inferred from model versus observed.

Supplementary Data 3 includes a xlsx file containing sediment flux data to select coastal deltas in the US over time as dam building progressed upstream of these deltas. We compare these fluxes to our modeled sediment yields extrapolated to the full basin upstream of each delta. The total basin sedimentation rates and fraction of basin trapped upstream from dams timeseries data are available at Zenodo[3].

Supplementary Note 4. Evidence that capacity loss is due to sedimentation.

We infer that surveyed capacity loss is due to sedimentation. This conclusion is supported by survey data noting an increase in the lowest, surveyed reservoir bottom elevation noted through time (refer to 'Generation Effort = Reservoir Sedimentation Surveys' on the Bureau of Reclamation's RISE database)[4]. Less commonly, sediment cores are collected from reservoirs; a recent example from the Bureau of Reclamation includes the coupling of sediment cores with a bathymetric survey at Pueblo Reservoir[5]. A study at Lake Englebright in California combined geophysical data, bathymetric surveys, and sediment cores[6–8] (e.g., Figure 5, Synder et al.[8]) and presents a longitudinal cross section of the pre- and post- reservoir profiles demonstrating vertical change in the lake floor along with sediment core stratigraphy. The Texas Water Development Board and Kansas Biological Survey have published several bathymetric surveys coupled with geophysical profiling of reservoir sediments or sediment coring[9,10]. The Lake

Travis (Texas) survey report provides an excellent example coupling sediment cores with geophysical survey data (e.g., Figure 8, Leber et al.[11]).

Supplementary Note 5. Sediment Yield and Multiple Linear Regression model comparison.

We compare model results from the sediment yield model ("SY" model in paper) and the multiple linear regression model ("MLR" model in paper) at unsurveyed reservoirs included in both models (see Figure 1 for schematic diagram). We perform this comparison at unsurveyed reservoirs located upstream from the 904 surveyed sites with permanent storage (n=27,691). The following query data and figures are exported by the "CompareSY_MLRAug25.m" function within the "ModelComboAnalysis_Aug2025.m" code (Supplementary Figure 1).

Query data for model comparison at n=27,691 sites:

MLR total capacity where SY model has no capacity in 2025: 0.1931 km^3

SY total capacity where MLR model has no capacity in 2025: 0.053215 km^3

MLR total reservoir sedimentation (2025): 8.6297 km^3

SY total reservoir sedimentation (2025): 7.9222 km^3

MLR total capacity where SY model has no capacity in 2050: 0.23936 km^3

SY total capacity where MLR model has no capacity in 2050: 0.050199 km^3

MLR total reservoir sedimentation (2050): 10.8009 km^3

SY, total reservoir sedimentation 2050: 10.1485 km^3

Supplementary Methods 1

To compare the Brown (1944) and Brune (1953)[12] trapping efficiencies, we utilize flow data from ResOps[13] reported at 298 reservoirs, available for varying time periods between 1980 and 2020. The ResOps dataset includes reservoirs in the Global Reservoir and Dam database (GRanD)[14]. Of the 298 reservoirs with inflow data, 203 are large reservoirs with design storage over 100 M m^3 and 95 are medium reservoirs with design storage between 6.2 and 100 M m^3.

The Brune trap efficiencies vary more from year to year, based upon the inflow. For long-term sedimentation, we are interested in the overall trend across the available data. For each reservoir, we compute the average trap efficiency for both Brune and Brown over the period of available inflow data (Supplementary Figure 11). Overall, 218 of 298 reservoirs (73%) produced Brown and Brune trap efficiencies within 5% of one another. 27 reservoirs report a mean Brown trap efficiency more than 5% higher than the corresponding mean Brune trap efficiency and 53 reservoirs report a mean Brune trap efficiency more than 5% higher than the corresponding mean Brown trap efficiency (Supplementary Table 3).

Holdover reservoirs retain storage from one season to the next, meaning that the overall data indicate frequently higher storage than annual inflows. These reservoirs may fill during very wet years. They also likely have lower outflows than inflows. For holdover reservoirs, 57 of 76 (75%) reservoirs produce Brown and Brune trap efficiencies within 5% of one another.

Seasonal storage reservoirs have mean-annual live storage (actual water storage, not just available capacity) that is less than the total annual inflow, meaning that they can fill each year and outflows often match inflows. ResOps data also include 2 reservoirs without annual live storage; we group these with the seasonal reservoirs. Small and medium sized reservoirs not included in the ResOps dataset are likely also dominated by seasonal storage reservoirs. For seasonal reservoirs, 161 of 222 (73%) reservoirs produce Brown and Brune trap efficiencies within 5% of one another.

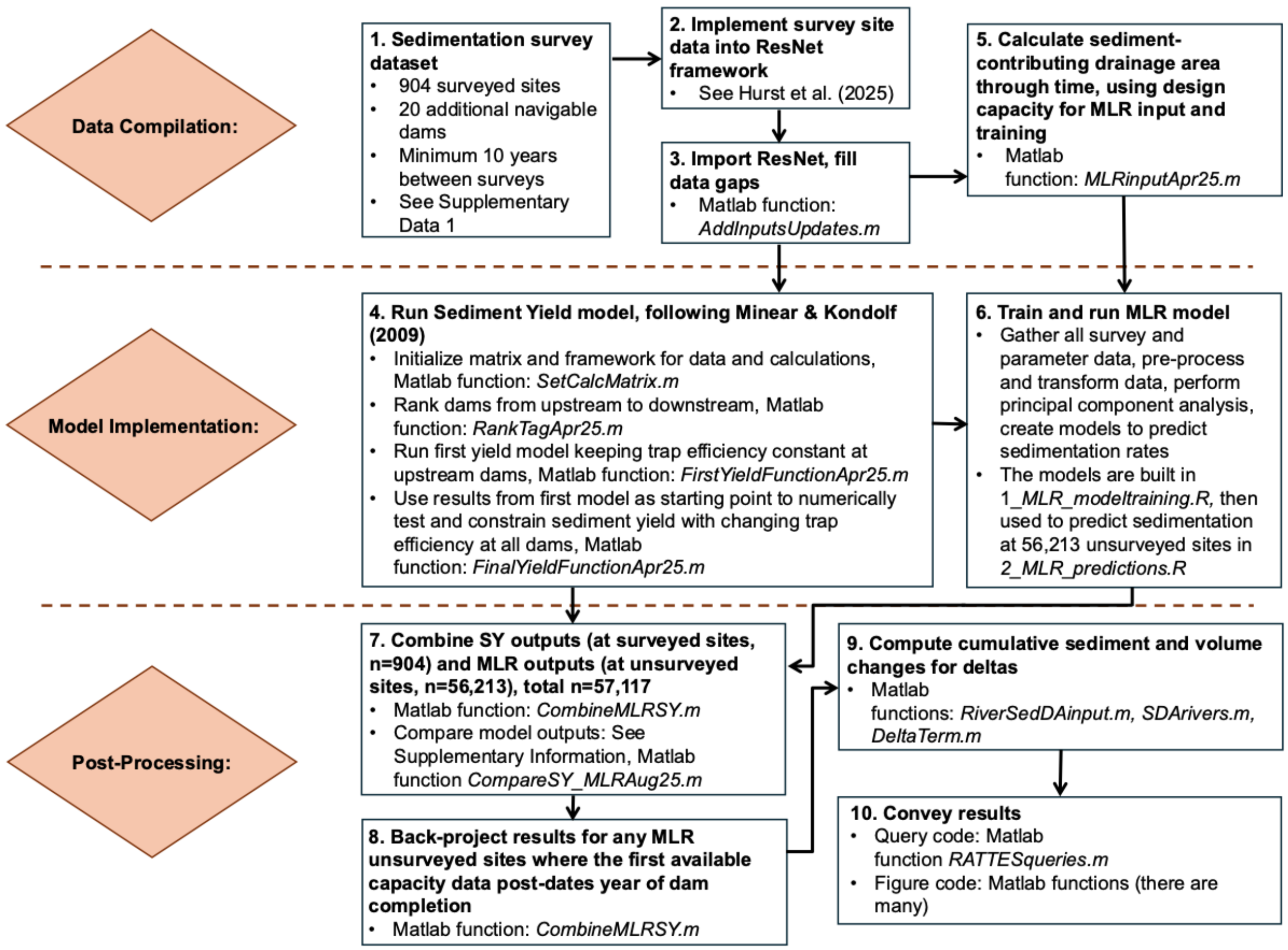

Supplementary Figure 1. Methods flowchart. Flowchart of methods and codes used to predict and convey RATTES results. The sand and clay runs follow the same workflow, but their corresponding kappa values are updated for Steps 4-10 (although not for model training). The various codes are available on Github (https://github.com/abbyeckland/rattes-naturecomms.git) and Zenodo[15].

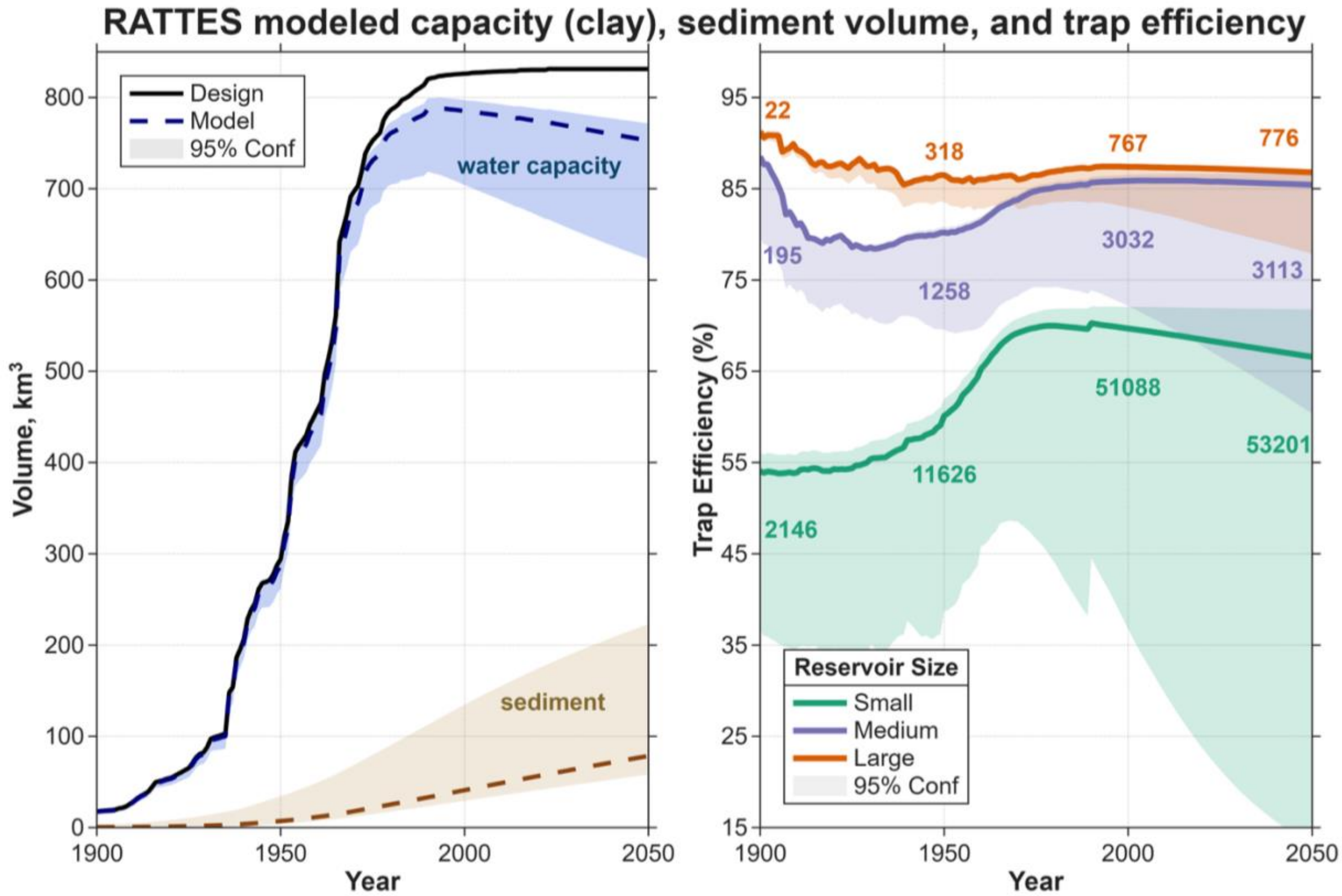

Supplementary Figure 2. RATTES model results (lines) with 95% confidence intervals (shading) for clay-sized sediment. (Left) Available water capacity (blue) and sediment volume (brown) from 1900 to 2050 for 904 surveyed sites and 56,213 unsurveyed reservoirs. (Right) Mean trap efficiency for reservoirs. Annotated numbers indicate the number of reservoirs in each category at years 1900, 1950, 2000, and 2050.

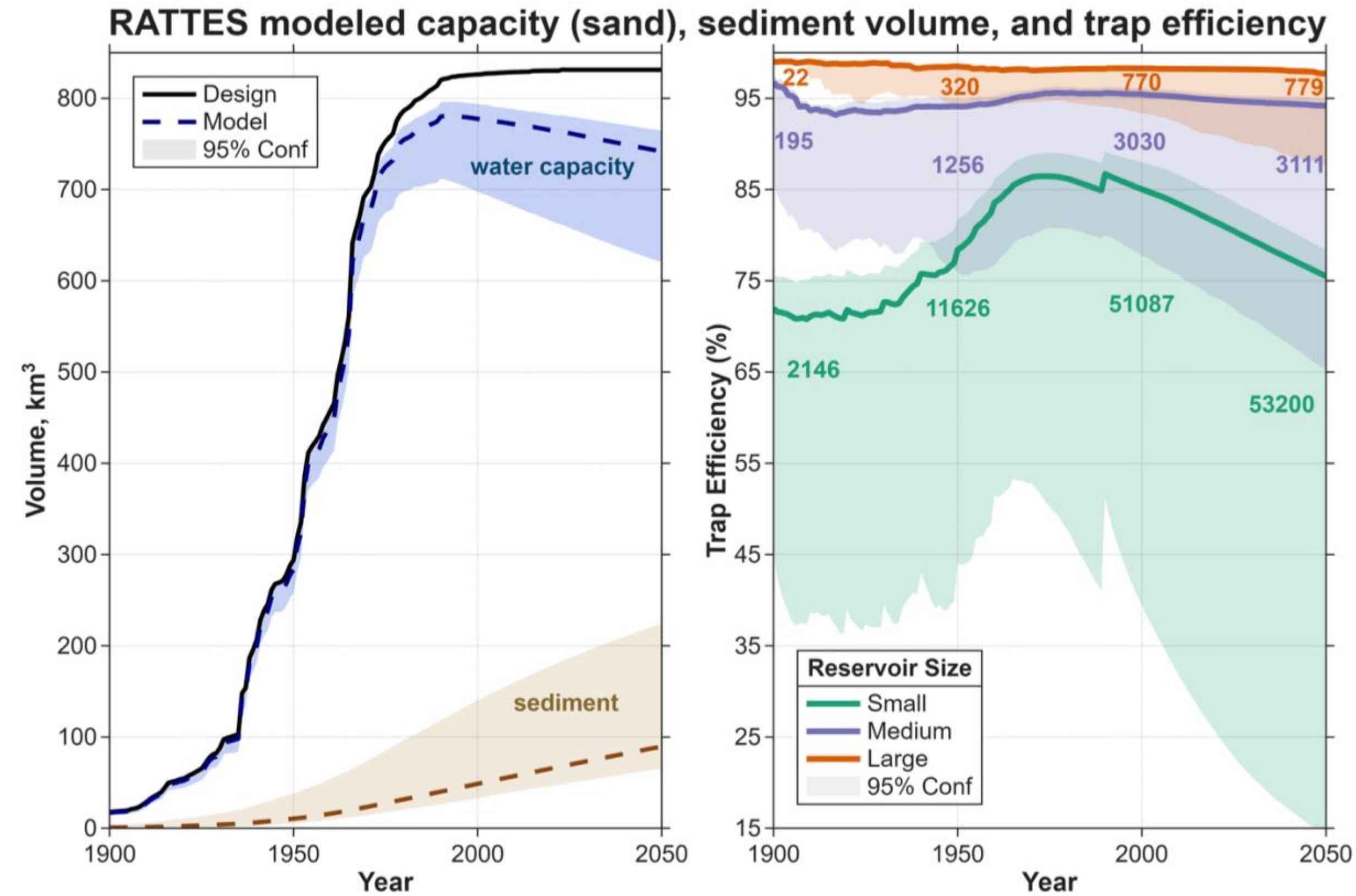

Supplementary Figure 3. RATTES model results (lines) with 95% confidence intervals (shading) for sand-sized sediment. (Left) Available water capacity (blue) and sediment volume (brown) from 1900 to 2050 for 904 surveyed sites and 56,213 unsurveyed reservoirs. (Right) Mean trap efficiency for reservoirs. Annotated numbers indicate the number of reservoirs in each category at years 1900, 1950, 2000, and 2050.

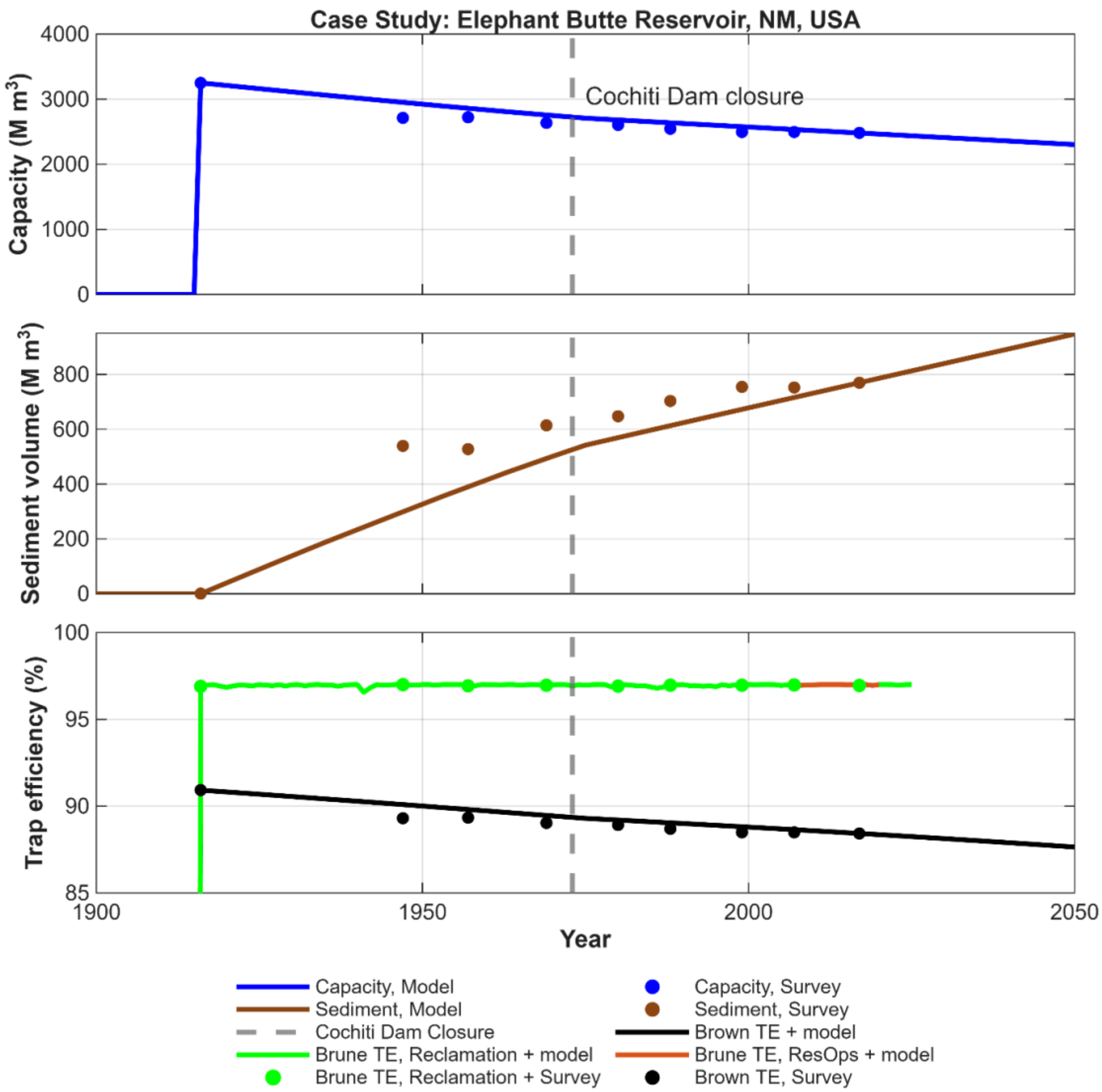

Supplementary Figure 4. Model results using the Sediment Yield model component at Elephant Butte Reservoir, New Mexico, US. (Top) Model capacity (blue line). Surveyed capacities are shown as blue dots. The model uses the first and last survey and does not use intermediate survey data. (Middle) Modeled sediment volume (brown line) and surveyed sediment volumes (brown points). (Bottom) Modeled trap efficiency using the Brown equation and modeled capacities (black line); black points are the Brown trap efficiency using surveyed capacities. Brune trap efficiencies using model capacities and inflow from Reclamation is shown as the green line and using available inflow from ResOps[13] is shown as the orange line (overlies green line). Brune trap efficiencies using surveyed capacity and mean annual inflow for survey years are shown as green points. See Figure 1 of Eckland et al.[16] for a map of the Elephant Butte Reservoir watershed, including the relative location of upstream dams, including Cochiti.

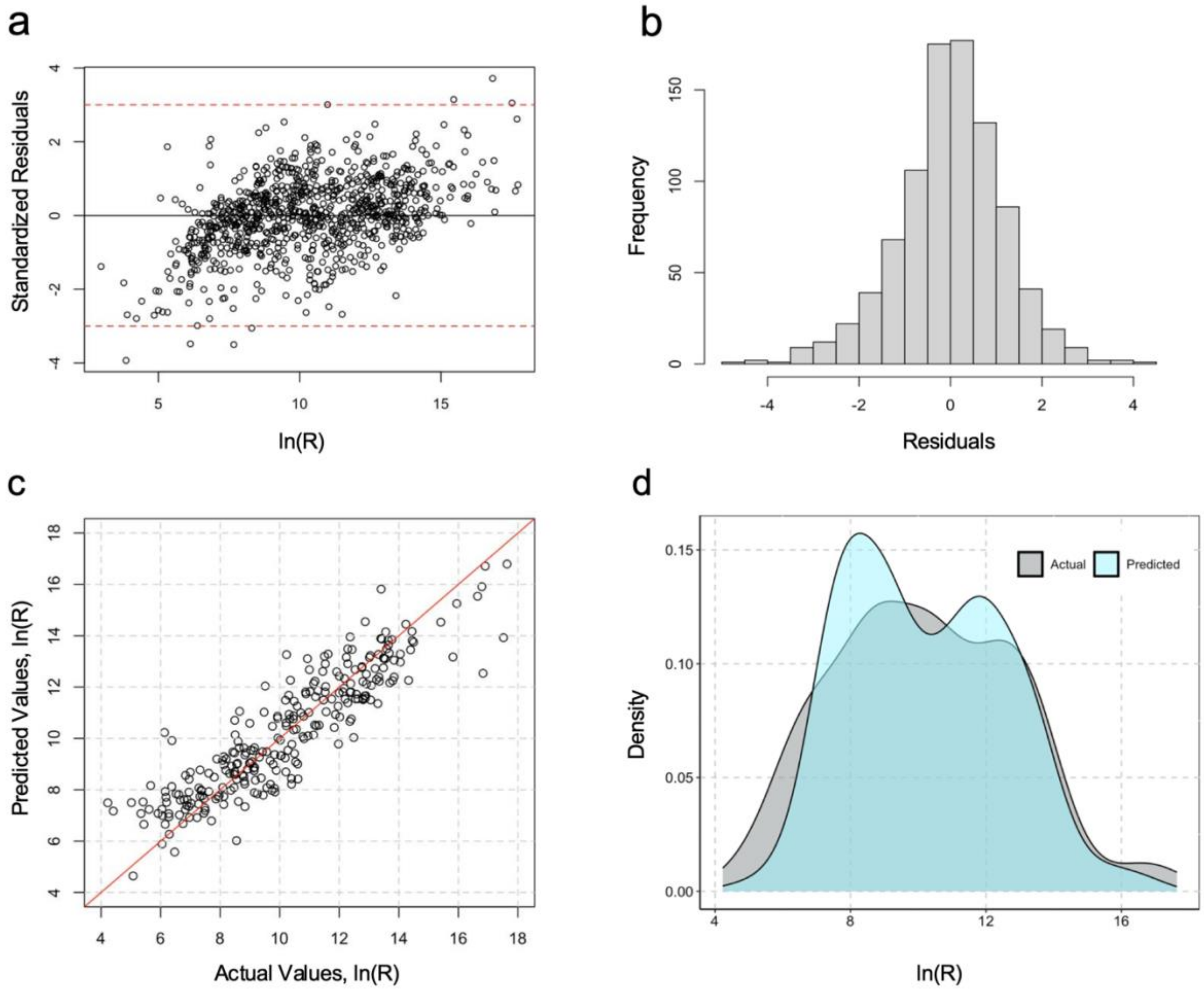

Supplementary Figure 5. MLR model performance diagnostics. A) Standardized residuals, B) histogram of residuals, C) actual versus predicted values of the natural log of the sedimentation rate, R, compared to 1:1 line, and D) probability density function curves for actual versus predicted values of ln(R). A and B contain results for all surveyed sites (n=904), while C and D contain results after splitting the data into training (70% or n= 633) and testing (30% or n= 271) sets.

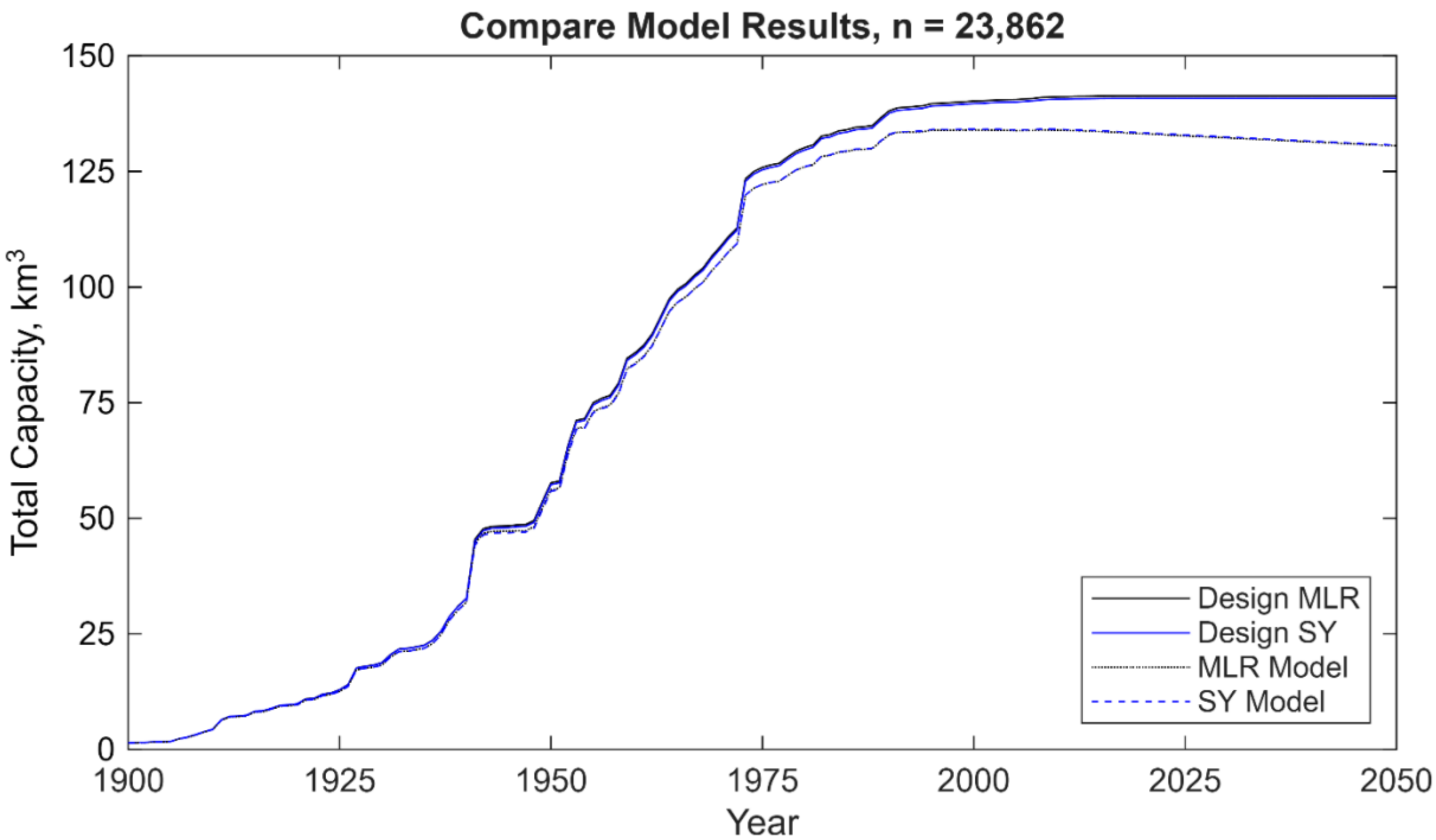

Supplementary Figure 6. Comparison of capacity results for SY and MLR model through time.

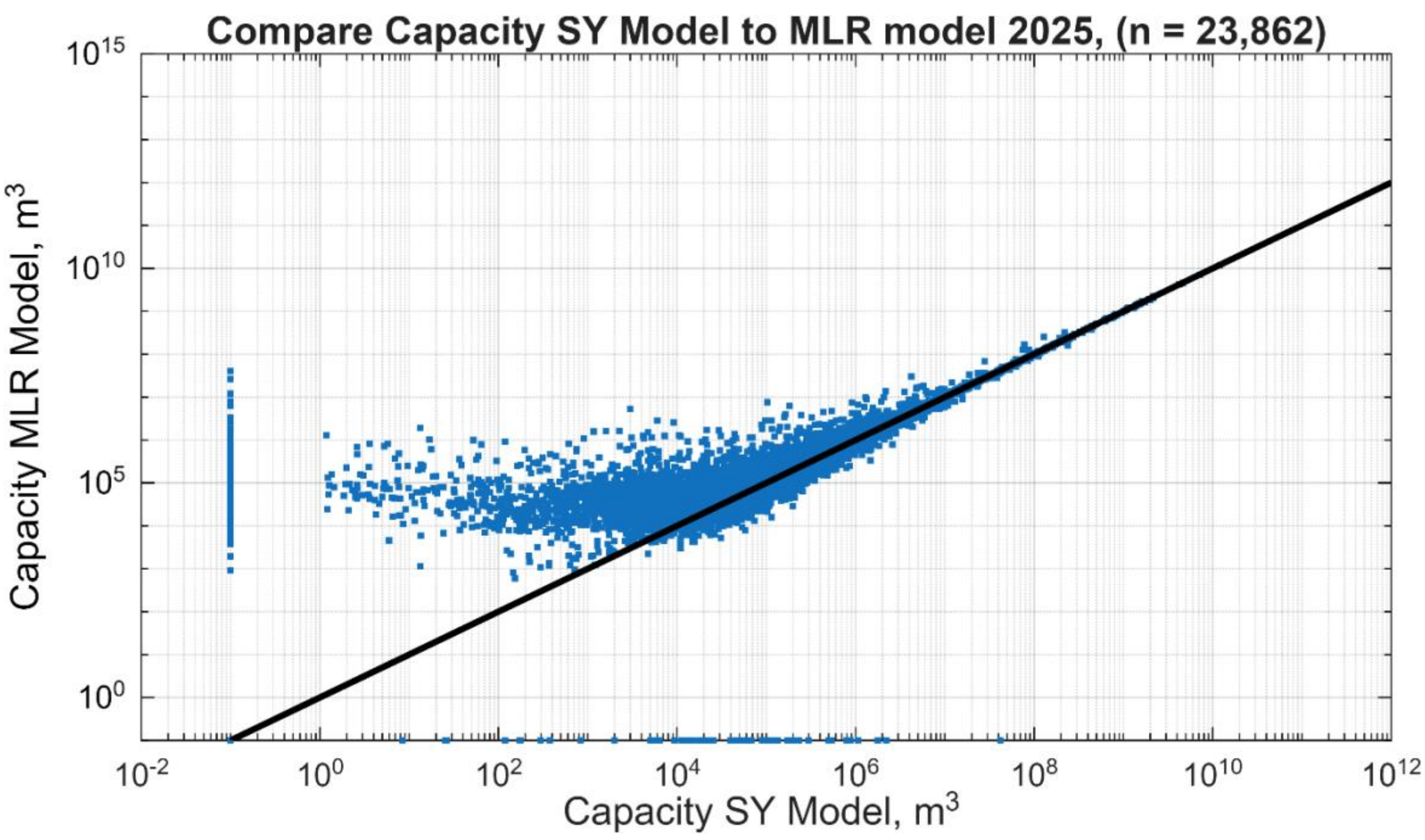

Supplementary Figure 7. Comparison of capacity estimates at unsurveyed reservoirs in both SY and MLR models, year 2025. Zeros values were converted to 10^{-1} for plotting purposes.

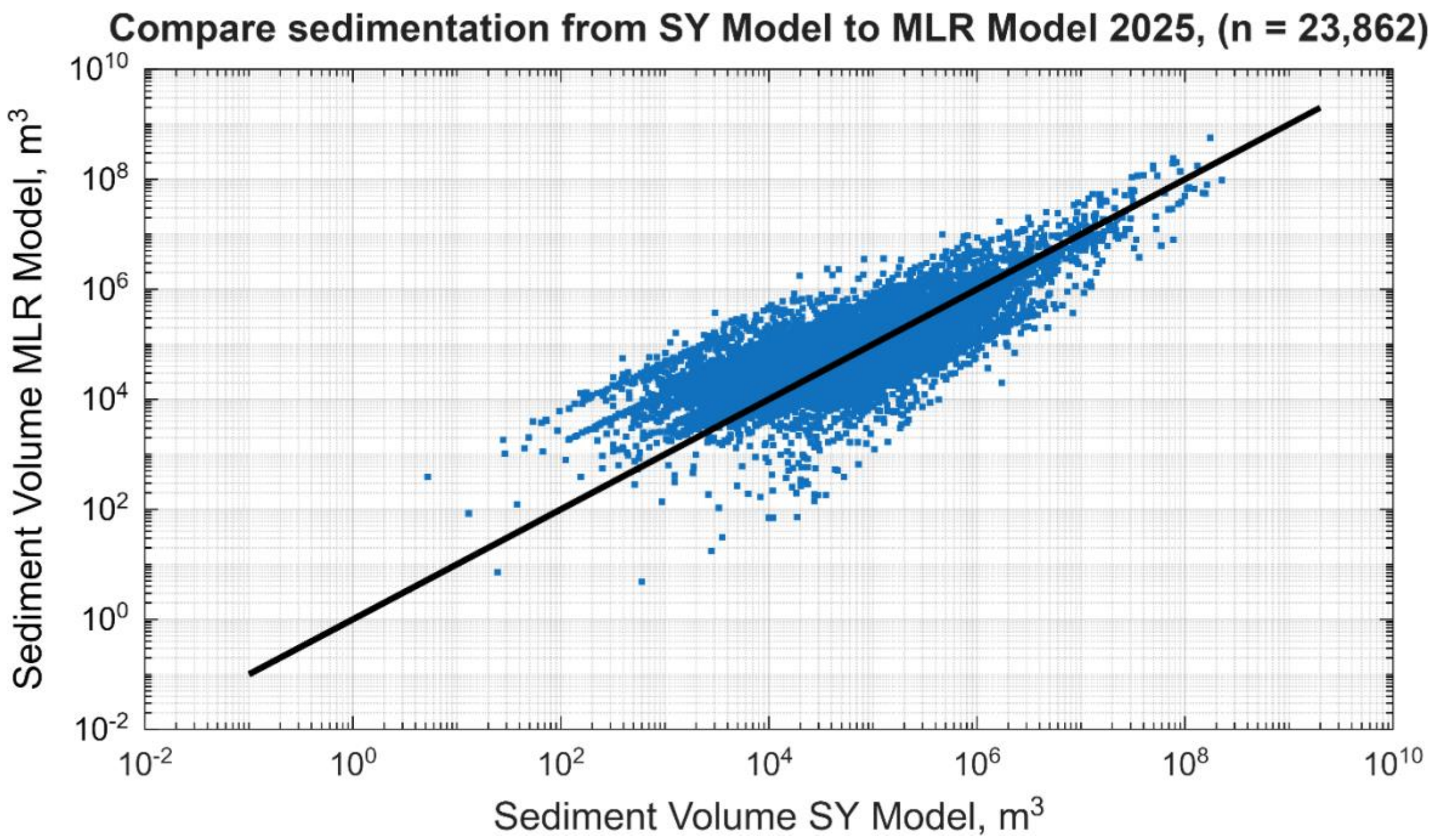

Supplementary Figure 8. Comparison of sedimentation estimates at unsurveyed reservoirs in both SY and MLR models, year 2025.

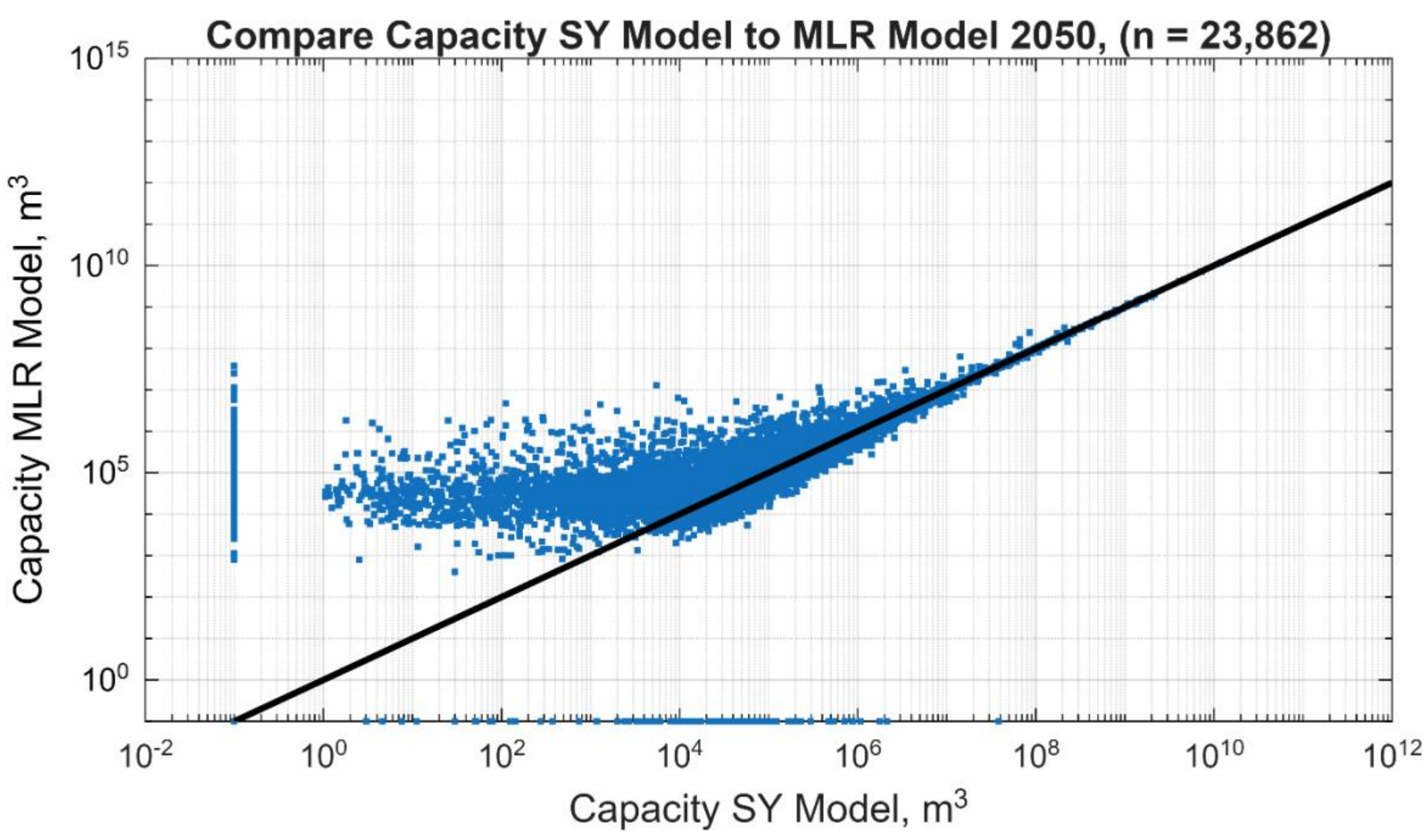

Supplementary Figure 9. Comparison of capacity estimates at unsurveyed reservoirs in both SY and MLR models, year 2050. Zeros values were converted to 10^{-1} for plotting purposes.

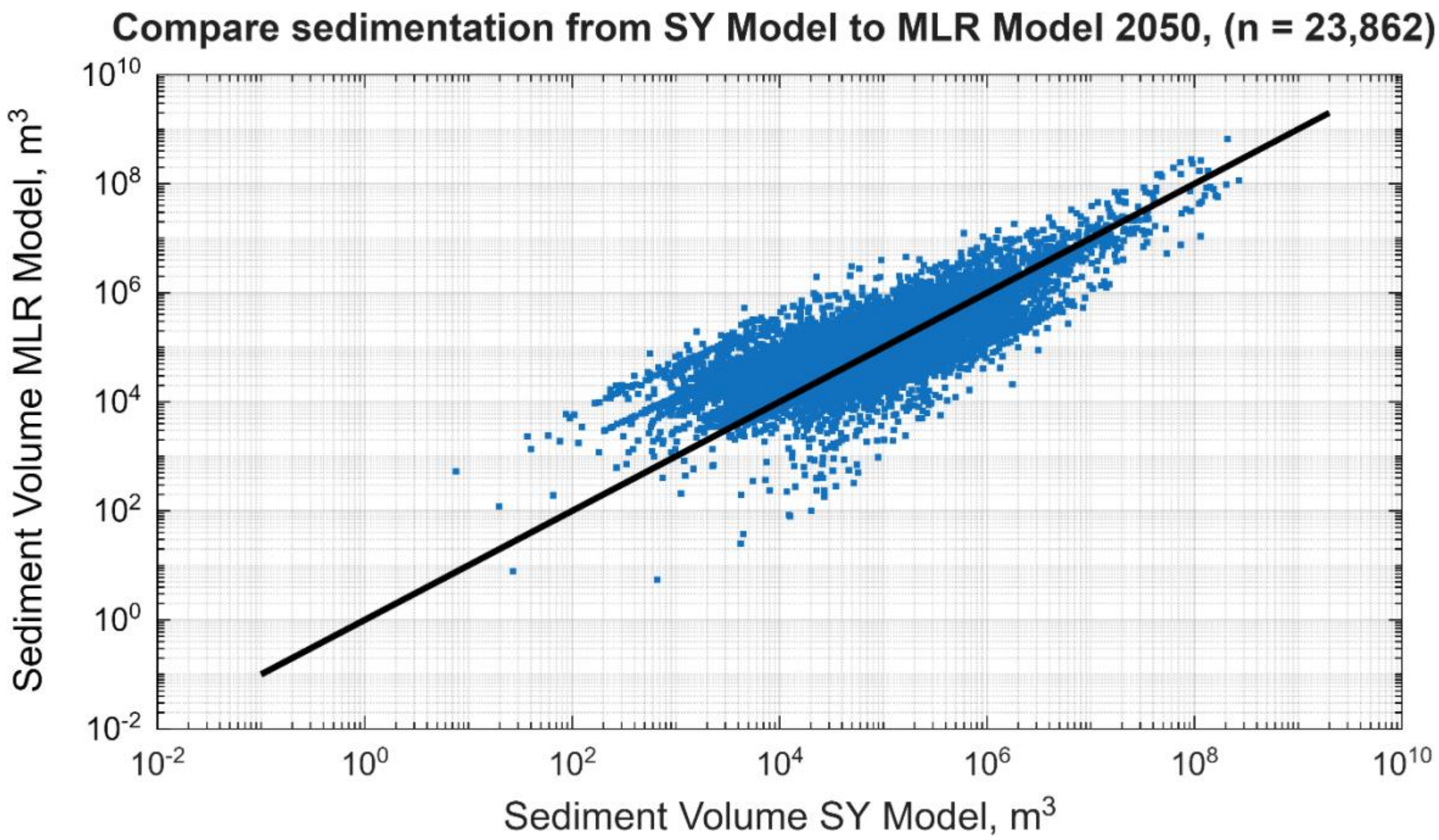

Supplementary Figure 10. Comparison of sedimentation estimates at unsurveyed reservoirs in both SY and MLR models, year 2050.

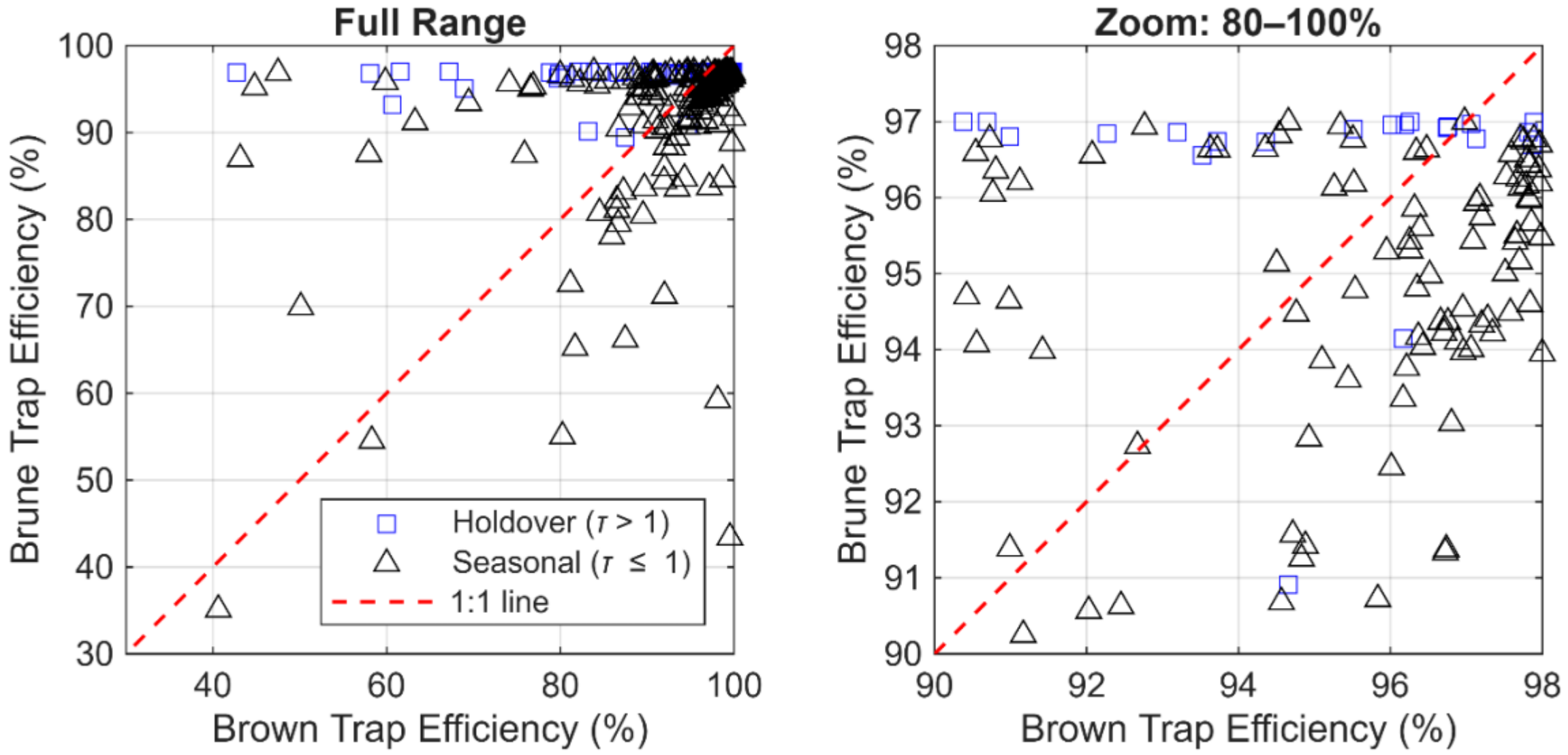

Supplementary Figure 11. Comparison between Brown (1944) and Brune (1953) trap efficiencies for 298 reservoirs reporting inflow data in ResOps[13]. Each point represents the mean Brown or Brune trap efficiency for years with available inflow data (Supplementary Methods 1).

Model ID	MLR Model	Number of parameters in model	Number of data points model is trained on	Residual standard error (RSE)	R-squared (R^2)
1	Final MLR	4	904	1.159	0.813
2	Extended MLR	11	904	0.999	0.862
3	Training MLR	4	633	1.154	0.809
4	Training Extended MLR	11	633	1.001	0.862
5	Reduced MLR	3	904	1.162	0.811
6	Reduced Training MLR	3	633	1.155	0.808

Supplementary Table 1. Comparison of MLR model performance metrics for the final model, the extended model, the training model, and the reduced model. See Github or Zenodo[15] for extended model summaries (https://github.com/abbyeckland/rattes-naturecomms.git).

**Sea level rise rate (mm/yr)**	**Applicable Date Range Used for Sea Level Rise Rate within RATTES model**	**Source note within Kemper et al., 2021**
1.35	1900 – 1970	Palmer et al., 2021
2.33	1971 – 2005	tide gages, https://psmsl.org/
3.69	2006 – 2010	Frederiske et al., 2020
4.9	2011 – 2020	SSP2-4.5 model scenario, 50th quantile
4.4	2021 – 2030	SSP2-4.5 model scenario, 50th quantile
4.9	2031 – 2040	SSP2-4.5 model scenario, 50th quantile
6.2	2041 - 2050	SSP2-4.5 model scenario, 50th quantile

Supplementary Table 2. Sea-level rise data, years 1900 to 2050. Data sourced from compiled data within Fox-Kemper et al.[17].

	Difference	Holdover Reservoirs (n=76)	Seasonal Reservoirs (n=222)
Brune TE higher than Brown TE (n=89)	>25	6	9
	10 to ≥ 25	8	12
	5 to ≥ 10	5	13
	0 to ≥ 5	12	24
Brown TE higher than Brune TE (n=209)	0 to ≥ 5	45	137
	5 to ≥ 10	0	18
	10 to ≥ 25	0	6
	>25	0	3

Supplementary Table 3. Comparison between Brune and Brown trapping efficiencies at holdover and seasonal reservoirs.

References

1. Hurst, A., Foster, M. & Eckland, A. The ResNet network of dams impounding storage reservoirs across the continental United States. *Sci. Data* (2025).
2. Hurst, A. ResNet. https://doi.org/10.5281/zenodo.15644268 (2025) doi:10.5281/zenodo.15644268.
3. Eckland, A. & Foster, M. RATTES Reservoir/Dam Impacts Upstream of Coastal Deltas (Years 1700-2050). https://doi.org/10.5281/zenodo.21692937 (2026) doi:10.5281/zenodo.21692937.
4. U.S. Bureau of Reclamation. RISE. *Reclamation Information Sharing Environment (RISE)* https://data.usbr.gov/catalog?filter=generationEffortId.50.
5. Varyu, D. R., Bradley, D. N., Cutrell, A. K. & Foster, M. A. *Pueblo Reservoir 2023 Sedimentation Survey and Sediment Coring*. 203 https://data.usbr.gov/rise/content-rise-public/rise/catalog-item/binary/PuebloReservoir_PuebloDam_CO00299_2023_Report_508.pdf (2023).
6. Snyder, N. P. *et al.* Reconstructing depositional processes and history from reservoir stratigraphy: Englebright Lake, Yuba River, northern California. *J. Geophys. Res. Earth Surf.* **111**, (2006).
7. Childs, J. R., Snyder, N. P. & Hampton, M. A. *Bathymetric and Geophysical Surveys of Englebright Lake, Yuba-Nevada Counties, California*. *Open-File Report* https://pubs.usgs.gov/publication/ofr03383 (2003) doi:10.3133/ofr03383.
8. Snyder, N. P. *et al.* Estimating accumulation rates and physical properties of sediment behind a dam: Englebright Lake, Yuba River, northern California. *Water Resour. Res.* **40**, (2004).
9. Texas Water Development Board. Completed Surveys & Data. https://www.twdb.texas.gov/surfacewater/surveys/completed/index.asp.

10. Kansas Biological Survey & Center for Ecological Research. Publications List. https://biosurvey.ku.edu (2026).

11. Leber, N., Holmquist, H., Iqbal, K., Duty, J. & Crouse, L. *Volumetric and Sedimentation Survey of Lake Travis, July-November 2019*. https://www.twdb.texas.gov/hydro_survey/travis/2019-11/Travis2019_FinalReport.pdf (2021).

12. Brune, 1953. Trap efficiency of reservoirs. *Eos Trans. Am. Geophys. Union* **34**, 407–418 (1953).

13. Steyaert, J. C., Condon, L. E., W. D. Turner, S. & Voisin, N. ResOpsUS, a dataset of historical reservoir operations in the contiguous United States. *Sci. Data* **9**, 34 (2022).

14. Lehner, B. *et al.* High-resolution mapping of the world's reservoirs and dams for sustainable river-flow management. *Front. Ecol. Environ.* **9**, 494–502 (2011).

15. abbyeckland. abbyeckland/rattes-naturecomms: RATTES full code. https://doi.org/10.5281/zenodo.21690856 (2026) doi:10.5281/zenodo.21690856.

16. Eckland, A. C., Overeem, I., Carlson, B. & Lininger, K. B. Accelerated Organic Carbon Burial Rates Reconstructed in Elephant Butte Reservoir, New Mexico During a Megadrought. *Water Resour. Res.* **61**, e2023WR035254 (2025).

17. Fox-Kemper, B. et al., 2021: Ocean, Cryosphere and Sea Level Change. In Climate Change 2021: The Physical Science Basis. Contribution of Working Group I to the Sixth Assessment Report of the Intergovernmental Panel on Climate Change [Masson-Delmotte, V. et al., (eds.)]. Cambridge University Press, Cambridge, United Kingdom and New York, NY, USA, pp. 1211–1362, doi:10.1017/9781009157896.011.
