# Supplementary material for: Reservoir sedimentation diminishes water storage and coastal delta resiliency
Source: Nat Commun. 2026 Aug 25;17:10164. doi: 10.1038/s41467-026-76986-3 (PMC13601615; doi:10.1038/s41467-026-76986-3)
Supplement: Supplementary file 2 [file 41467_2026_76986_MOESM2_ESM.pdf]

Description of Additional Supplementary Files

File Name: Supplementary Data 1

Description: Site data used to regenerate ResNet.

File Name: Supplementary Data 2

Description: Delta data.

File Name: Supplementary Data 3

Description: Sediment fluxes to select deltas through time: Inferred from model versus observed.
