# Supplementary material for: Reservoir sedimentation diminishes water storage and coastal delta resiliency
Source: Nat Commun. 2026 Aug 25;17:10164. doi: 10.1038/s41467-026-76986-3 (PMC13601615; doi:10.1038/s41467-026-76986-3)
Supplement: Supplementary file 3 [file 41467_2026_76986_MOESM6_ESM.pdf]

nature portfolio

Peer Review File

Reservoir sedimentation diminishes water storage and coastal delta resiliency

Corresponding Author: Dr Abigail Eckland

This file contains all reviewer reports in order by version, followed by all author rebuttals in order by version.

Version 0:

Reviewer comments:

Reviewer #1

(Remarks to the Author)
I consistently reviewed the paper entitled "Reservoir sedimentation reduces water storage and coastal delta resiliency" by Abigail C. Eckland, Melissa A. Foster, Aaron A. Hurst, Mussie Beyene, and Irina Overeem. The manuscript Ref. No. is NCOMMS-26-014058-T.

The key take-home messages can be summarized as follows:
A. The importance of reservoir sedimentation consequences of frequently overlooked small and mid-size reservoirs is highlighted
B. Sedimentation rates from other literature sources are often overestimated
C. The GIS and ‘big data’ approach is promising
D. The withheld sediment upstream of river deltas no longer reach them, favoring delta degradation
E. Without upstream reservoir trapping sediment (unreasonable hypothesis), the transport material could compensate sea-level rise (without indicating the resulting delta degradation actually happening)

The paper deals with the generalized assessment of reservoir sedimentation by predicting the capacity loss and sedimentation rates of 57,117 water storage reservoirs of all sizes across the continental United States, combined with the appraisal of this effect on river mouth delta aggradation and sea-level rise.

The combination of these two spatially (and to a certain extend temporally) distinct processes (upstream reservoir sedimentation AND coastal river mouth aggradation) is not completely novel (several studies on beach replenishment in Italy and Spain to recover spaces for touristic activities close to retreating rivers mouths deltas), but still presents an distinctive feature of the presented work.

Taking into account not only the largest, well documented reservoirs in the study, but all size reservoirs adds an additional unique feature that sets it apart from previously published works.

The highly complex reservoir sedimentation processes are largely simplified using the sole Brown equation (1944!), no distinction is made with respect to incoming sediment yield variability (changes in the upstream watershed, grain size distribution, distinction between bed and suspended load, flood event based sediment input, turbidity currents, reservoir delta aggradation, sediment routing or flushing, etc.). It would be interesting to show graphically the evolution of the reduction in reservoir trap efficiency for some selected reservoirs (e.g., small, mdi-size and large) form first impoundment to 2050. Not a single case study reservoir with available data is used to validate the results, no RATTES parameter calibration attempt can be identified, nor uncertainty regionalization across such a large geographic domain.

In the present study, fortunately the reduction in reservoir trap efficiency due to progressing reservoir sedimentation through time is taken into account. The applied simplification is deemed necessary in the presented overall approach, but should be more accurately cited in the text and supported by relevant literature. Citations and literature on reservoir sedimentation processes, including sediment transit could be enriched.

A clear distinction to be made (this could be identified as a major flaw) is the missing sediment size degradation during transit along the river network, from the coarser upstream part of the watershed to the finer material (wash load) in the delta

region. It is not clear from the paper, how the modelling of the sediment transit time or sediment routing through the fluvial/reservoir system from source to sea is performed.

Another point to discuss is the extrapolation to the year 2050. The reviewer could not find relevant information on this extrapolation method: how is climate change taken into account, which IPCC scenario (e.g., RCP8.5, RCP4.5) is used? The reviewer was unable to identify this in the reservoir sedimentation 20250 prediction. With respect to estimated 20250 sea-level (SLR) rise figures, the used climate scenario could be identified in the supplementary data source literature (SSP2-4.5 model scenario).

Nevertheless, the reviewer acknowledge the rationality of the approach, the use of apparent good quality and well referenced data.

There are no real conclusions in the manuscript, and the presented solutions to delta degradation by acting on the terminal reservoirs (working on sediment trap efficiency, venting, flushing, dam removal, etc.) are not deeply presented and supported by examples from literature.

Data interpretation is deemed robust, valid and reliable. With respect to the numerous modelling result presentation, the reviewer would prefer to see some pie charts or similar graphs, not just bare figures and percentages.

(Remarks on code availability)

Reviewer #2

(Remarks to the Author)
review of reservoir sedimentation reduces water storage and coastal delta resiliency by Jaap Nienhuis

I have read and assessed the study of Eckland et al, who apply their RATTES model to study reservoir sedimentation across ~60,000 US water reservoirs.

I enjoyed reading the paper and I think it has great potential. I especialy like figure 2, which combines past trends with future projections. I did spot some things that will need to be discussed before publication.

As a major comment is that the paper could benefit from more physical insights into its results or discussion. For example: I expect a large difference in the results from sediment grain size (mud vs sand).

The time component is (somewhat ironically) not discussed much. A nice study from Rodriguez et al (Nature Communications 2020) showed increases in coastal sedimentation across the US despite dams, likely because of erosion downstream of dams. Nittrouer and Viparelli (Nature Geoscience 2014) also make a case of long time lags for sand. Wang et al (Nature Communications 2025) find short time lags.

A third physical component that I miss is a comparison with (now many) studies that look at along river sediment fluxes (e.g., Dethier et al Science 2022) How do (trends) from these studies compare to your findings?

Also, when reading the paper, it occured to me only very late that the study never actually looks at river sediment fluxes itself. If I understand correctly, the brown equation is modified with different drainage areas to simulate downstream effects of dams on other dams (small note that the Atot_alpha in equation 5 should then probably be rewritten as A* to avoid confusion?).

In addition to these major comments, I have a set of minor suggestions below. Broadly however, I think the paper is novel in it aims and I like the connection to deltas (although, as the authors ackowledge, some of the resulting aggradation deficits are unrealistic). I recommend moderate revisions and I look forward to the authors' response.

Minor
L29: break up introduction into a few more paragraphs.
L51: what is meant with "living"?
L55: "conduct numerical models" -> simulations?

L79: perhaps phrase it the other way around? a relationship between environmental predictors to sedimentation rates? (as in: the objective of the MLR is to obtain sedimentation rates?)

L89: what are the units in this equation? and what do the parameters mean?

L102: I've been looking for a while but I can't seem to find 5 squares in the figure?

L104: very cool results!

L114: capacity loss of water I assume (and not sediment?). A bit more explanaiton of this key sentence would be good.

L132: this scaling is remarkable. What is the underlying cause? The large increase in TE for larger reservoirs? Or simply the dicharge and sediment flux scaling itself? For clarity of writing: i would move this result to a new paragraph where you focus on the effect of small vs large reservoirs. Right now it reads like an afterthought of the literature comparison.

L138: is the overestimation also something you see in your sedimentation data? It would be good to confirm.

L143: this paragraph, and some others, are a mix of discussion and results and extracting the key message is therefore a bit difficult.

L215: have trapped?

L256: this sentence repeats the same message as the previous sentence?

L272: just to confirm, this would mean that (a part of) these 7% would then flow to the delta?

a bit broad comment: sections like L193, L292, L302, L315 feel a bit detached from your primary model results. Some of these sections don't really build on the (many) insights you can get from your model. At the same time, I can think of some others ideas that aren't discussed much (see major comment above).

conclusion could be phrased more from the perspective of new scientific knowledge (and less about what the model can do?)

L543: a schematic figure of the model would be helpful here.

L650: A bit more information is needed to assess the uncertainty of the SYSR and SSUR models as I do not fully understand this. For example, uncertainty from a trap efficiency equation could be passed forward to RATTES uncertainty, right? Or uncertainty can be assessed by training the model on 2/3rds of the data and then applying it to the remaining 1/3rd?

(Remarks on code availability)

Reviewer #3

(Remarks to the Author)
What are the noteworthy results?
Leveraging a comprehensive database of 57,117 water-storage reservoirs across the continental United States, the authors quantify the volume of sediment trapped behind dams and the associated loss of reservoir storage capacity. They translate this sediment sequestration into equivalent delta aggradation rates and evaluate whether these rates could keep pace with eustatic and relative sea-level rise. The manuscript further provides estimates of the fraction of sediment stored behind terminal dams, highlighting a potential focus for sediment-management interventions.

Will the work be of significance to the field and related fields? How does it compare to the established literature? If the work is not original, please provide relevant references.
The United States is among the most heavily dammed regions globally. This study appears to be among the first high-resolution, contiguous US-wide, reservoir-by-reservoir estimates of sediment storage and associated capacity loss that explicitly includes small and mid-sized reservoirs. The findings are significant for quantifying anthropogenic alteration of sediment transfer from source to sink, especially in the context of climate-driven hydrologic change and accelerating sea-level rise. The results should be useful for river–delta restoration planning and for prioritizing sediment-management interventions and/or dam removal. More broadly, because sand and gravel are among the most extracted and economically important materials by volume, translating trapped sediment volumes into an approximate economic value (where feasible) could be particularly impactful for resource-management discussions.

Does the work support the conclusions and claims, or is additional evidence needed?
Conclusions and claims are generally supported by the data and results. A central assumption in estimating sediment volume is that reservoir capacity loss through time is an adequate proxy for sediment trapping and accumulation. It would strengthen the study to ground-truth this key assumption using lake-bottom cores from a subset of reservoirs with documented capacity surveys. Where available, such cores could provide a more direct constraint on sediment thickness (and accumulation rates) since dam construction and help evaluate potential biases from factors other than sedimentation (e.g., survey method changes, operational drawdown effects, or bathymetric uncertainty) that can also contribute to apparent capacity loss over time.

Are there any flaws in the data analysis, interpretation and conclusions? Do these prohibit publication or require revision?
Data analysis and interpretation appear robust, and the conclusions are generally supported by the results. One issue that merits more explicit discussion is dam removal. In general, since the early 2000s the United States has been removing more dams than it is building. How does this trend affect (or potentially complicate) the authors' interpretation of reduced reservoir trap efficiency due to dam aging (lines 252–256)? In particular, do dam removals and decommissioning introduce a survivorship bias in the remaining reservoir population, and how is this accounted for in RATTES?

Is the methodology sound? Does the work meet the expected standards in your field?
The authors present a clear methodology, supported by detailed supplementary information, and key assumptions are stated throughout. Overall, the approach appears sound and consistent with expected standards in the field. One aspect that deserves more thorough treatment is the impact of sediment compaction. Although the authors briefly mention compaction (line 621), it should be explained in greater detail, including how compaction is represented in converting between deposited sediment volume, thickness, and mass. For example, if an exponential relationship between compaction and deposit age is assumed, the manuscript should justify the functional form and parameter choices, and discuss the sensitivity of results to these assumptions.

Is there enough detail provided in the methods for the work to be reproduced?
The authors provide sufficient methodological detail for the work to be reproduced. The code is available on GitHub, and the underlying data are provided in an Excel database, enabling others to replicate the analyses and results.

Suggestions for minor editing of the manuscript:
Line 11: Add the word “surface” after third word (i.e., “in surface reservoirs”)
Line 19: Clarify that this is total sediment (61.3 GT)
Line 29: Add the word “surface” after third word
Line 59: Replace third word (“from”) with “in previous”
Line 333: Replace fourth word (“accurate”) with “useful”
Line 667: Replace “depth” with “thickness”
Line 678-680: Check grammar. Sense is not clear.

(Remarks on code availability)

Version 1:

Reviewer comments:

Reviewer #1

(Remarks to the Author)
The authors consistently modified the manuscript according to the three reviewers' comments.
The rebuttal gives responses to major comments as well as to individual reviews.
I highly appreciate the endurance and clarity of the response, and modifications and additions in the manuscript's text and figures.
I have no objection for publication at this stage.

(Remarks on code availability)

Reviewer #2

(Remarks to the Author)
Review by Jaap Nienhuis.

I have now re-read the manuscript and I think it has been greatly improved. I thank the authors for their answers to my earlier review and I have only a few remaining remarks:

One thing I would like to clarify about my earlier review, reading the author's subsequent response, is that I realize that the RATTES model does not explicitly resolve fluvial sediment transport. Nevertheless, the presented results still would benefit from the context of (spatial/temporal) observations of the effect of dams on sediment fluxes to deltas, especially if implications are drawn for delta resiliency. For example: the authors present a sediment flux (sediment volume over time) trapped by dams in figure 3a, and further present several hints about temporal aspects of sediment trapping (Fig 5b). How does this flux compare to observations of sediment flux increase across North American rivers reported by Dethier (2022), or the Mississippi work by Nittrouer & Viparelli (2012)? Is there any relation between the (predicted) sediment storage in dams and the (observed) sediment flux increase to deltas? Such a comparison (perhaps only qualitatively in the discussion) would provide significant strength (or necessary nuance) to your conclusions about delta resilience.

minor comments:
L130: it would be useful here to say explicitly which (anthropogenic) parameters are important. The formula (1) only has geomorphological/hydrological characteristics (drainage area, specific discharge), but this is probably not what you mean.
L174: I find this a very important conclusion of your study. Perhaps highlight it more in the abstract/conclusion?

(Remarks on code availability)

Reviewer #3

(Remarks to the Author)
I thank the authors for their careful and detailed responses to my comments and other reviewers'. Overall, I am satisfied with the level of efort in addressing the reviewers' comments and the amount of revision. The additional text clarifying the methods and underlying concepts will help readers understand the manuscript, and the new schematic in Fig. 1 is helpful for conveying the modeling approach, the governing equations, and the analyses. My three principal concerns from the first round were each engaged directly, as summarized below.

1. Reservoir capacity loss as a proxy for sediment accumulation
The authors have strengthened the justification for this central assumption (lines 588-598), citing bathymetric reports that document rising reservoir-bottom elevations over time, together with Bureau of Reclamation cores and Texas Water Development Board geophysical surveys at selected sites. I appreciate also the added screening of the survey data (removing records where capacity increased and requiring a minimum 10-year inter-survey interval), which addresses part of the survey-method and dam-raise artifacts I raised. This is a reasonable and field-standard treatment, and I accept the authors' point that a quantitative, core-based ground-truth is not feasible at the national scale of this study.

One suggestion: if at all possible, it would meaningfully strengthen reader confidence if the authors could show, in the supplementary material, at least one example sediment core paired with the corresponding capacity-loss survey at the same reservoir. Even a single well-documented case (e.g., a Bureau of Reclamation site) presented as a supplementary figure would directly illustrate that measured capacity loss corresponds to accumulated sediment thickness. I leave this to the authors' discretion given data availability.

2. Dam removal and survivorship bias
This is addressed well. The author clarify that RATTES is not an analysis of a fixed reservoir population but a dynamically evolving network in which reservoirs enter (construction) and exit (removal) on an annual basis, with the sediment-contributing drainage area re-adjusted for downstream structures after each removal (lines 69-70, 552). This design directly defuses my survivorship-bias concern, since the model simulates the actual time-evolving population rather than filtering to surviving reservoirs. The Elwha example (Figure 5B) is a clear illustration, and the context that ~2,350 documented removals are small relative to >57,000 standing dams is helpful. I also appreciate the authors' take about what the volumetric framework cannot do: namely, route the sediment pulse released downstream following a removal. That limitation is appropriately stated and is acceptable for the scope of this paper.

3. Sediment compaction
The treatment is improved and the response is made in good faith. The authors explain that compaction is captured implicitly because sedimentation rates are derived by differencing repeat bathymetric surveys, and they have forced the time parameter (T) into the model as an empirical proxy for time-dependent processes including compaction, supported by a sensitivity test in which removing T changes the predicted volume by roughly 10% while R^2 remains stable (lines 593-598, 694-698). I accept the argument that an explicit compaction function is not supportable at the national scale.

Two small points would further tighten this section. First, the single reported mass (61.3 Gt), which underpins the prominent comparison that US reservoir sediment exceeds the annual global fluvial load by roughly seven times, rests on a single constant bulk density (960 kg/m^3). Because density varies with sediment age and grain size (the very compaction effect under discussion), I recommend the authors attach a brief sensitivity range to that mass figure so the headline comparison carries an explicit uncertainty. Second, the sensitivity values cited in the rebuttal (predicted storage changing from 20.5 to 22.6 km^3 when T is removed) do not obviously reconcile with the 63.8 km^3 total reported in the main text; if these refer only to the unsurveyed/MLR component, a one-line clarification in the text or supplement would avoid confusion.

Minor Points
All seven of my first-round minor edit have been incorporated.

(Remarks on code availability)

Open Access This Peer Review File is licensed under a Creative Commons Attribution 4.0 International License, which permits use, sharing, adaptation, distribution and reproduction in any medium or format, as long as you give appropriate credit to the original author(s) and the source, provide a link to the Creative Commons license, and indicate if changes were made.
In cases where reviewers are anonymous, credit should be given to 'Anonymous Referee' and the source.
The images or other third party material in this Peer Review File are included in the article's Creative Commons license, unless indicated otherwise in a credit line to the material. If material is not included in the article's Creative Commons license and your intended use is not permitted by statutory regulation or exceeds the permitted use, you will need to obtain permission directly from the copyright holder.
To view a copy of this license, visit https://creativecommons.org/licenses/by/4.0/

Responses to Major Comments

Stronger physical grounding of sediment transport processes
RATTES is a volumetric reservoir capacity-loss model trained on bathymetric survey data. It is not a traditional sediment transport model capable of quantifying sediment fluxes in discrete river reaches since it does not capture point-source sediment contributions and cannot model sediment transport associated with specific flood events. We now more clearly stated in the revised Introduction the benefits and limitations of our modeling approach—that we can track sediment volumes and reservoir storage capacity through time as dams are constructed and removed—but that we cannot calculate sediment fluxes in individual river reaches or account for rapid, event-based sedimentation (lines 92-99). By adding a schematic diagram with example calculations in revised Figure 1, we better clarify our approach, driving equations, and analyses.

RATTES' strength is that it is the first volumetric estimate of sediment storage in the entire US water storage network, trained on repeat bathymetric survey data, enabling us to better understand the impacts of dams on national-scale stream systems. RATTES includes thousands of small reservoirs in upper watersheds that serve as important sediment traps and have been largely ignored in past studies. More than 66% of the estimated reservoir sedimentation occurs at reservoirs with bathymetric surveys, increasing confidence in our volumetric model results. Further, this is the first model to quantify the volume of stranded sediment stored within small to large water storage reservoirs across entire river basins located upstream from coastal deltas across the contiguous United States.

Grain size effects
We agree with the reviewers that the sediment volume trapped in a reservoir depends on the grain size, which impacts the mode of sediment transport and particle settling rates. Our original model employed a silt-sized sediment assumption, employed by other researchers[1,2] and design standards[3,4]. To supplement and bolster our findings with respect to grain size, we employed sand and clay sediment sizes in the final RATTES model to produce endmember solutions. This gave us error bounds, allowing the calculated trap efficiency to vary as a function of grain size, in addition to the changing active capacity and drainage area. The total estimated volume of sediment contained in US reservoirs with permanent storage in 2025 decreases from 69.6 km^3 assuming sand, to 63.8 km^3 assuming silt, and to 60.2 km^3 assuming clay (lines 138-142). The addition of the clay and sand-size sediment endmembers improves the model; we thank the reviewers for their feedback.

Because our study covers the entire US, it was not feasible to obtain measured grain size data for all incoming sediment through time—especially given that the incoming

*sediment size changes through time with the emplacement of additional upstream dams. For background, we also note that we explored using the US D_{50} dataset tied to the NHD flowlines[5]. However, these data indicated that almost all reservoirs had a D_{50} of sand-sized material. Based on **very** limited sediment core data obtained by the Bureau of Reclamation and other studies, these estimates appeared too coarse to represent the entire volume of sediment stored in reservoirs. We were concerned that applying sand-size sediment across the US would result in over-estimates of reservoir sedimentation. Thus, we retain the silt-sized assumed for the "middle-of-the-road" estimates but supply the clay- and sand endmember volumes in the main text, with full output provided in [Zenodo]() and additional code provided in [GitHub]().*

Assumptions underlying 2050 projections

The RATTES model assumes that the parameters controlling future sedimentation mimics past sedimentation. This does not mean that sedimentation rates are constant with time. They depend upon changes in the sediment-contributing drainage area and changes to the other parameters in the RATTES model. This is true of the assumptions at any year for the model.

Moving beyond measured data parameters and into the future, we assume that there are no new dams emplaced past current day and no additional dams removed. Further, parameter data continue to update as time passes, trap efficiency wanes, and sedimentation progresses throughout the dam network, like all other years in the model (see lines 718-720, Equations 1-7). Similar to the recent study by Das et al.[6], our study shows that anthropogenic impacts to watersheds exert the primary control on reservoir sedimentation (Supplementary Table 1). Overall, the number of new dam emplacements and dam removals is very small relative to all 57,000 existing dams. Since the sediment-contributing drainage area is our strongest sedimentation predictor[7], based on the in-place dam network, we extended the model projections beyond present day. Because our model cannot include parameters such as changing hydrology or the increased prevalence of fire, we did not want to project past trends too far into the future. However, we decided that projecting the near future was an important metric to provide as a baseline to inform future sedimentation trends.

Reservoir capacity loss reflects reservoir sedimentation

The reduction in available reservoir capacity (or storage) is inferred as the volume of reservoir sediment in numerous studies[1,8–10]. Available storage capacity refers to the total volume of a reservoir that could be filled with water. This differs from live storage, which is the actual volume of water stored in a reservoir at any given time. Live storage will vary due to inflows and operations and can be reduced drastically during periods of drought.

In the revised Methods, we add text (lines 588-598) to explain the assumption that reservoir capacity change is driven by sediment accumulation and supported it with references to reports and previous articles focused on best practices to measure reservoir sedimentation[1–4]. This assumption is supported by sediment cores obtained by the Bureau of Reclamation at select sites, as well as geophysical surveys documenting the properties of reservoir sediment at select sites by the Texas Water Development Board[11,12]. Further, the Bureau of Reclamation survey reports document the lowest reservoir bottom elevation in each survey and demonstrate how this elevation has increased through time due to sedimentation.

Compaction

Lane and Koelzer[13] related particle size distribution of reservoir sediment to the initial density at the time of deposition and Miller[14] found that density changes through time due to wetting and drying, as the result of compaction. Thus, sediment density is a function of reservoir age and initial particle size distribution.

We do not have operational data for all the reservoirs in the US to explicitly constrain compaction. Instead, we have our training dataset of surveyed reservoirs, which report the volume of capacity lost. Our model assumes that future operations mimic past operations, allowing us to project capacity loss volumes into the future based upon past trends, including any compaction that previously occurred at surveyed sites. We chose to primarily report volumes, rather than sediment density, to minimize the uncertainty compaction introduces in these conversions. We report only one cumulative mass of sediment in the paper, 61.3 GT, using the reported density value of 960 kg m^{-3} from Dendy et al.[1] and Minear and Kondolf[2], solely for the purpose of comparing the mass of stored sediment with the global fluvial sediment load delivered to oceans annually of 8.5 GT[15].

When exploring dozens of parameters to include in the RATTES model, we found that reservoir age was the second most important parameters of 11 for predicting reservoir sedimentation[7]. Reservoir age implicitly incorporates the effect of compaction as the observed sedimentation rates are lower at older reservoirs, even with similar upstream sediment-contributing drainage areas. Reservoir age explains 7.2% of the model variance, which is overall very low compared to the sediment-contributing drainage area, which explains 66.8% of the variance. Ultimately, we decided to limit RATTES to the top 4 parameters, because these data are readily available, allowing the RATTES framework to be transferred to other regions. However, recognizing the potential importance of reservoir age and how this likely serves as a proxy for compaction, we forced the final RATTES model to include reservoir age as a parameter. The final RATTES formulation explains 81% of the model variance with readily accessible data.

In the revised Methods, we discuss how compaction is implicitly incorporated in the observed sedimentation data, since any compaction of previously deposited sediments is measured when differencing reservoir bathymetric surveys collected at different points in time (592-597). Explicitly including compaction in our models using an empirical formula would require more information on reservoir operations and grain size than we are able to obtain from existing databases, which we clarify in the text.

Trap Efficiency

We now explicitly justify our use of the Brown[16] (1944) trap efficiency equation in the revised Introduction. We employed the Brown equation due to a lack of annual inflow data for all 57,117 reservoirs in our study, impeding the use of the Brune[17] (1953) equation and others[18–20]. For the large scale of our study, we believe the Brown equation is appropriate to establish first-order estimates of past, present, and future sedimentation across US reservoirs. Minear and Kondolf[2], whose approach we apply nationwide for surveyed reservoirs, also used the Brown equation for this reason.

However, annualized inflow data for varying periods between 1980 and 2020 are available for 298 primarily large reservoirs included in the GRanD dataset[21] through the ResOps database[22]. For these reservoirs, we provide a comparison between the Brune and Brown trap efficiencies in the Supplementary Methods 1. The trap efficiencies vary from year to year based on the inflow, but over the period of record, 218 of the 298 reservoirs produced mean Brown and Brune trap efficiency values within 5% of one another. Within the 5% threshold, 182 of the 218 reservoirs produced a higher mean Brown trap efficiency than the corresponding mean Brune trap efficiency. However outside of the 5% threshold, 53 reservoirs reported a higher mean Brune trap efficiency than Brown trap efficiency, compared to 27 reservoirs reporting a mean Brown trap efficiency more than 5% greater than the corresponding Brune mean trap efficiency. Overall, we view the two trap efficiency equations as producing similar results, within the magnitude of the RATTES model uncertainty.

Dam removal

The number of dam removals across the United States is currently 2,350 according to American Rivers. This is an incredibly small value compared to the more than 57,000 in-place dams. Dam removals are included in the model when the source ResNet dataset[23] notes a dam removal year. The model then removes this structure and the sediment-contributing drainage area is adjusted for downstream structures (clarified in line 552). Again, RATTES is a volumetric reservoir capacity-loss model trained on bathymetric survey data and is not a sediment transport model capable of quantifying sediment fluxes in discrete river reaches. As such, it does not explicitly track sediment volumes released due to dam removal and potential impacts on the downstream reservoirs. Further, many inland dam removals likely include dredging or sediment management plans to control sediment releases to downstream systems; we do not

have these data to incorporate in the model. Our model adjusts the sediment-contributing drainage area following dam removal, providing a larger volume of sediment to downstream structures following dam removal. In addition, the largest US dam removals on the Elwha and Klamath River systems drain directly to the coast and thus their removal did not supply sediment to other dams remaining in the national network.

Responses to Individual Reviewers

Reviewer #1 (Remarks to the Author):

I consistently reviewed the paper entitled "Reservoir sedimentation reduces water storage and coastal delta resiliency" by Abigail C. Eckland, Melissa A. Foster, Aaron A. Hurst, Mussie Beyene, and Irina Overeem. The manuscript Ref. No. is NCOMMS-26-014058-T.

The key take-home messages can be summarized as follows:
A. The importance of reservoir sedimentation consequences of frequently overlooked small and mid-size reservoirs is highlighted
B. Sedimentation rates from other literature sources are often overestimated
C. The GIS and 'big data' approach is promising
D. The withheld sediment upstream of river deltas no longer reach them, favoring delta degradation
E. Without upstream reservoir trapping sediment (unreasonable hypothesis), the transport material could compensate sea-level rise (without indicating the resulting delta degradation actually happening)

- *Thank you for the feedback. To address point E, we have clarified our language when comparing reservoir sediment volumes with potential delta aggradation. The volume of sediment stored upstream behind reservoirs is extremely large and without reservoir trapping, much of this sediment would eventually reach coastal systems. We acknowledge that deltas are degrading due to subsidence and other factors and that sea level rise varies locally. Both can be true. We provide the delta aggradation analysis simply to relate reservoir volumes to delta-specific metrics. We have clarified our language and avoided portraying this comparison as a simple cause and effect (lines 19-21, 83-88, 250-263).*

The paper deals with the generalized assessment of reservoir sedimentation by predicting the capacity loss and sedimentation rates of 57,117 water storage reservoirs of all sizes across the continental United States, combined with the appraisal of this effect on river mouth delta aggradation and sea-level rise.

The combination of these two spatially (and to a certain extent temporally) distinct processes (upstream reservoir sedimentation AND coastal river mouth aggradation) is not completely novel (several studies on beach replenishment in Italy and Spain to

recover spaces for touristic activities close to retreating rivers mouths deltas), but still presents a distinctive feature of the presented work.

- *Thank you for bringing up the studies in Italy and Spain, we have reviewed these studies and considered their findings in our revision.*

Taking into account not only the largest, well documented reservoirs in the study, but all size reservoirs adds an additional unique feature that sets it apart from previously published works.

The highly complex reservoir sedimentation processes are largely simplified using the sole Brown equation (1944!), no distinction is made with respect to incoming sediment yield variability (changes in the upstream watershed, grain size distribution, distinction between bed and suspended load, flood event based sediment input, turbidity currents, reservoir delta aggradation, sediment routing or flushing, etc.). It would be interesting to show graphically the evolution of the reduction in reservoir trap efficiency for some selected reservoirs (e.g., small, mid-size, and large) form first impoundment to 2050. Not a single case study reservoir with available data is used to validate the results, no RATTES parameter calibration attempt can be identified, nor uncertainty regionalization across such a large geographic domain.

- *Thank you for these thoughtful comments. Please see the above discussion of trap efficiency in the “Responses to Major Comments” section above. We added a figure plotting trap efficiency at small, medium, and large reservoirs through time (Figure 3B); we include additional figures applying sand and clay sized sediment endmember solutions in Supplementary Figures 2 and 3. We also added the comparison between Brune and Brown trapping efficiency to address your comment (Supplementary Methods 1).*

- *On sediment yield variability – we agree the model indeed does not account for temporal changes in the upstream basin with regards to grain size, partitioning of suspended and bed load supply, flood event-based sediment inputs, turbidity currents, reservoir delta aggradation, sediment routing or flushing, etc. We clarified the model limitations in the text (lines 92-99). Reservoir surveys are not conducted rapidly enough to capture event-based sedimentation (except in special rare cases), so it is not possible to obtain these data or to predict these patterns for the 57,117 reservoirs in our study. The reservoir sedimentation survey data we applied in the model spanned a minimum of 10 years between surveys: 1) to increase the likelihood of a flood occurring that transports and deposits a discernable amount of sediment in the reservoir between surveys; 2) to capture any potential reservoir sediment management activities between reservoir surveys, although we avoided these cases where possible. Nonetheless, sediment removal is very rare and flushing often does not remove large volumes of sediment.*

- *On reservoir delta aggradation – if the reservoir delta elevation is within the total reservoir elevation capacity, then delta sedimentation is captured in the survey data*

and in the reported total capacity loss. If delta aggradation extends farther upstream, then our results would underestimate actual reservoir sediment volumes.

- *We also provided a case study plot illustrating capacity, sedimentation, and trap efficiency results over time for a surveyed reservoir, Elephant Butte, in Supplementary Figure 4 to illustrate how sedimentation and trap efficiency decreases over time as a reservoir infills with sediment. It also illustrates how the sedimentation rate changes instantaneously at a dam when a dam is built upstream, which reduces the sediment-contributing drainage area.*

- *Model parameterization is discussed in more detail in the Methods in section "Multiple linear regression model for unsurveyed reservoirs" (lines 680-705). We also reference Dr. Eckland's PhD dissertation[7], where model parameterization is discussed in depth. We added Supplementary Figure 5, which illustrates how the MLR model performs well. We also added Supplementary Table 1 summarizing the model performance and error statistics for the fully parameterized model (Extended MLR) versus the 4-parameter RATTES model (Final MLR). Full model summaries are also provided on Github.*

- *Finally, on the uncertainty regionalization, we apply the same uncertainty bounds to all reservoirs in the study since we have built a single model to predict reservoir sedimentation for the entire country. We experimented with separate models for distinct geographic regions but found that a single model performs remarkably well on a national scale. We plan to create regional models in future publications to highlight parameters that better predict localized reservoir sedimentation, which varies regionally with climate and geology. We also provide 1:1 plots for 23,862 unsurveyed reservoirs, comparing the results between the sediment yield approach, based on Minear and Kondolf[2], and the multiple linear regression model (Supplementary Figures 6-10).*

In the present study, fortunately the reduction in reservoir trap efficiency due to progressing reservoir sedimentation through time is taken into account. The applied simplification is deemed necessary in the presented overall approach, but should be more accurately cited in the text and supported by relevant literature. Citations and literature on reservoir sedimentation processes, including sediment transit could be enriched.

- *Thank you, we agree this is an important advance compared to previous estimations of continent-wide trapping. We bolstered our discussion of sediment transport and deposition processes and their relation to trap efficiency in the text in lines 62-82 of the Introduction and added relevant references.*

A clear distinction to be made (this could be identified as a major flaw) is the missing sediment size degradation during transit along the river network, from the coarser upstream part of the watershed to the finer material (wash load) in the delta region. It is

not clear from the paper, how the modelling of the sediment transit time or sediment routing through the fluvial/reservoir system from source to sea is performed.

- *Indeed, sediment grain size in fluvial systems typically decreases from headwater regions to the coast, most notably for bed material. This is why we obtained modeled median bed material grain size values (D_{50}) for rivers inflowing into each reservoir in our previous study that developed ResNet[23]; these data was originally modeled by Abeshu et al.[5] (2022) but incorporated in the ResNet output. We expected to see more significant grain size degradation as distance to the coast decreased. However, we found that most of the D_{50} values of bed material remained sand regardless of distance to the coast. In the Brown equation, sand is represented by a single coefficient value, k, of 1.0; thus, grain size fining with distance downstream is not captured by k, which decreases only if the mode fines to silt (k=0.1) or clay (k=0.046).*

- *Additionally, the bed material load of the river inflowing into the reservoir is not a reflection of the mode of sediment that is actually retained in the reservoir, the latter of which is a combination of bed material and settled wash load. The combined resulting grainsize D_{50} turns out to be typically silt[3]. Thus, for the original manuscript model, we implemented a constant grain size mode of silt to calculate trap efficiency for all reservoirs. Below in the response to reviewer 2, we outline additional runs we conducted for the revised manuscript that bracket the k value in the Brown equation to obtain upper and lower bounds for sedimentation and capacity losses across all study reservoirs.*

Another point to discuss is the extrapolation to the year 2050. The reviewer could not find relevant information on this extrapolation method: how is climate change taken into account, which IPCC scenario (e.g., RCP8.5, RCP4.5) is used? The reviewer was unable to identify this in the reservoir sedimentation 20250 prediction. With respect to estimated 2025 sea-level (SLR) rise figures, the used climate scenario could be identified in the supplementary data source literature (SSP2-4.5 model scenario).

- *Please see the discussion of extrapolation to year 2050 in the "Responses to Major Comments" section above.*

- *Our models unfortunately cannot directly account for climate change because they are trained using data collected largely over the 20th century, and emphasized in the early 20th century when reservoirs were more commonly surveyed during the age of dam construction. Extrapolation to the year 2050 is performed the same way as the model predicts sedimentation for any year occurring after the year of the second survey (or for the prediction dataset, any year following the year it was first surveyed), by first updating the capacity, then using the new capacity to calculate a new trap efficiency which is applied to the sedimentation prediction for the following year, and so on and so forth. Accounting for climate-driven change in sediment transport is out of scope of this paper. There are indications that the hydrological cycle intensifies, but sediment transport appears to be decreasing in temperate and*

arid regions[24], while increasing in tropical and Arctic regions[25]. Whereas effects of climate are hard to disentangle from damming[26], our approach disentangles the trapping effects.

- *To calculate the delta aggradation metrics, we do account for accelerating projected sea level rise. Supplementary Table 2 contains the sea level rise values for each time period and the source/scenario. For example, the SSP2-4.5 model scenario, 50th quantile is utilized for years 2011 to 2050 and the sea-level rise rate over that time varies from 4.9 to 6.2 mm/yr.*

Nevertheless, the reviewer acknowledge the rationality of the approach, the use of apparent good quality and well referenced data.

- *Thanks!*

There are no real conclusions in the manuscript, and the presented solutions to delta degradation by acting on the terminal reservoirs (working on sediment trap efficiency, venting, flushing, dam removal, etc.) are not deeply presented and supported by examples from literature.

- *We expanded the discussion on how removal of the Elwha River dams has led to progradation of the Elwha delta within just 5 years of the removals (see lines 310-313).*

- *We revised the conclusion by highlighting the main scientific findings of the paper and emphasized broader implications. See lines 350-372.*

Data interpretation is deemed robust, valid and reliable. With respect to the numerous modelling result presentation, the reviewer would prefer to see some pie charts or similar graphs, not just bare figures and percentages.

- *We have added several more figures to help visualize the results. See revised Figures 3 and 4 and Supplementary Figures 2-5, which include histograms, results from a case study, Elephant Butte Reservoir, and expanded Figure 3, which includes the trap efficiency over time for different sized reservoirs.*

Reviewer #2 (Remarks to the Author):

Review of reservoir sedimentation reduces water storage and coastal delta resiliency by Jaap Nienhuis

I have read and assessed the study of Eckland et al, who apply their RATTES model to study reservoir sedimentation across ~60,000 US water reservoirs.

I enjoyed reading the paper and I think it has great potential. I especialy like figure 2, which combines past trends with future projections. I did spot some things that will need to be discussed before publication.

As a major comment is that the paper could benefit from more physical insights into its results or discussion. For example: I expect a large difference in the results from sediment grain size (mud vs sand).

- *Thank you for bringing this up. The Brown trap efficiency equation (Equation 5) includes a coefficient, k, representing the sediment particle size. For the original paper, we computed trap efficiencies assuming silt as the dominant grain size retained in reservoirs (k=0.1). We made this assumption due to a lack of grain size data for all reservoirs in our study. Our assumption is supported by a much smaller subset of reservoir sediment data showing that silt is the predominant grain size class retained in reservoirs*[3]*.*

- *ResNet provides modeled median sediment grain size (D_{50}) data for each reservoir in our study*[23]*. These data were obtained from the NHD flowline intersecting the NID dam and represents the modeled bed sediment grain size of the inflowing river*[5]*; thus, it does not necessarily reflect the mixed bedload and settled washload grain size mode that is retained upstream of a dam. For the reservoirs in our study, most D_{50} values of bed material represent sand. However, observational data that silt is the predominant mode retained in reservoir sediment, so assuming sand as the predominant mode of sediment retained in reservoirs would overestimate sediment trapping.*

- *For the revised manuscript, we ran two additional simulations to obtain upper and lower bounds for trap efficiency by assuming all sediment retained is sand (k=1.0) and that all sediment retained is clay (k=0.046). The main simulation still follows the assumption that silt is dominant (k=0.1), so sediment volumes reported in the text remain largely unchanged. Supplementary Figures 2 and 3 show the upper and lower bounds obtained from the two additional model runs ran with clay and sand-based kappa values. These new results are also available on Zenodo.*

The time component is (somewhat ironically) not discussed much. A nice study from Rodriguez et al (Nature Communications 2020) showed increases in coastal sedimentation across the US despite dams, likely because of erosion downstream of dams. Nittrouer and Viparelli (Nature Geoscience 2014) also make a case of long time lags for sand. Wang et al (Nature Communications 2025) find short time lags.

- *We simply cannot account for sediment transport time lags since our modeling approach does not track sediment particles from source to sink, it tracks sedimentation volumetrically based on measured basin sediment yield rates. We clarify this in lines 92-99 of the revised Introduction to avoid confusion and to reiterate our goal for the paper, which is to estimate the location and magnitude of reservoir sediment trapped upstream of coastal deltas, and to help visualize this magnitude by converting the volume to potential total and annual delta aggradation rates.*

A third physical component that I miss is a comparison with (now many) studies that look at along river sediment fluxes (e.g., Dethier et al Science 2022) How do (trends) from these studies compare to your findings?

- *We do not calculate along-river sediment fluxes in this study. First, since we only know the volume of sediment deposited in a reservoir over time, we calculate a volumetric sedimentation rate. From this, we calculate an annual sedimentation rate. To calculate sediment flux into a reservoir, we would have to multiply the sedimentation rate by the inverse of the dam's trap efficiency. In many cases where there are multiple tributaries into a single reservoir, it would be difficult to determine the sediment flux of individual rivers using a single sediment flux value; this also puts a strong emphasis on the trap efficiency equation which, as pointed out by the reviewers, is limited in its utility since it does not include a unit of inflow.*

- *Second, we cannot calculate sediment flux downstream of a terminal dam all the way to the coast since we have no explicit data to constrain the sediment yield rate of the landscape below a terminal dam, so we would not have a way of quantifying the additional contribution of sediment from downstream tributaries or erosion below a dam. We are keenly aware of the limits of the Brown trap efficiency equation, as pointed out by the reviewers, thus we do not perceive it would be valid to calculate and present sediment fluxes in the present paper.*

Also, when reading the paper, it occured to me only very late that the study never actually looks at river sediment fluxes itself. If I understand correctly, the brown equation is modified with different drainage areas to simulate downstream effects of dams on other dams (small note that the Atot_alpha in equation 5 should then probably be rewritten as A* to avoid confusion?).

- *On river sediment fluxes – see the response immediately above the present comment.*

- *On the Brown equation (Equation 5), Atot_alpha is the correct variable name. In this TE equation, we use the total drainage area since this is a parameter we obtain from ResNet. The effect of upstream dams on downstream dams is accounted for in Equations 3 and 4 which compute the sediment-contributing drainage area (Ased_alpha, time) and the effective sediment-contributing drainage area (Asedeff_alpha,time).*

In addition to these major comments, I have a set of minor suggestions below. Broadly however, I think the paper is novel in it aims and I like the connection to deltas (although, as the authors acknowledge, some of the resulting aggradation deficits are unrealistic). I recommend moderate revisions and I look forward to the authors' response.

- *We agree, the "potential delta aggradation" is for exploratory purposes. We now better discuss that there are limiting assumptions; a uniform delta trapping*

coefficient of 30% (which we know is less accurate for small delta systems), a dependency on the mapped delta area (which we defend as it is commonly used in similar studies), and ignoring the fact that once substantial aggradation may have occurred, subsequent flooding would not inundate the delta floodplain (our aggradation is a gross number). However, we maintain that this is a useful metric for comparison to other controls on delta elevation, like subsidence.

Minor

L29: break up introduction into a few more paragraphs.

- *Broke up paragraph 1 in the introduction into 2 (line 38). Inserted a new paragraph discussing more background on trap efficiency (lines 62-70).*

L51: what is meant with "living"?

- *As in can be reproduced in the future as its input datasets are updated.*

L55: "conduct numerical models" -> simulations?

- *We updated the wording in line 54.*

L79: perhaps phrase it the other way around? a relationship between environmental predictors to sedimentation rates? (as in: the objective of the MLR is to obtain sedimentation rates?)

- *Indeed, we rephrased this sentence per the reviewer's suggestion (lines 107-109).*

L89: what are the units in this equation? and what do the parameters mean?

- *The units and parameter definitions are provided in the paragraph above Equation 1, in lines 109-114.*

L102: I've been looking for a while but I can't seem to find 5 squares in the figure?

- *There are 5 small squares above each selected delta (black triangle with numbers). These squares represent the same dams that are shown in Figure 5. We wanted to highlight spatially where the largest sediment-trapping reservoirs are located in the continental interior. We have selected a set of 16 deltas to show in Figure 2 and Table 1 because including the top 5 sediment-trapping reservoirs for every delta in the US (36) would get too busy. See Supplementary Data 2 for the full delta table, which includes the ResNet ShortID for the top 5 sediment trapping reservoirs above all 36 deltas.*

L104: very cool results!

- *Thanks!*

L114: capacity loss of water I assume (and not sediment?). A bit more explanaiton of this key sentence would be good.

- *Clarified that water storage capacity loss is driven by sedimentation. Also see discussion above in the "Responses to Major Comments" section.*

L132: this scaling is remarkable. What is the underlying cause? The large increase in TE for larger reservoirs? Or simply the dicharge and sediment flux scaling itself? For clarity of writing: i would move this result to a new paragraph where you focus on the effect of small vs large reservoirs. Right now it reads like an afterthought of the literature comparison.

- *Great point. We moved the comparison of sedimentation effects in small versus large reservoirs to the second paragraph in this section (lines 148-158), now before the comparison with previous studies (lines 174-184). We also added revised Figure 4 to show the differences in sedimentation impacts between small, medium, and large reservoirs.*

L138: is the overestimation also something you see in your sedimentation data? It would be good to confirm.

- *The MLR model actually underestimates sedimentation in large reservoirs. Splitting the survey data into training (70%) and test (30%) datasets allowed us to evaluate model performance. For reservoirs with very high sedimentation rates (typically large reservoirs), the predicted sedimentation rate is lower than the observed sedimentation rate (Supplementary Figure 5C and D). Note that R is the natural logarithm of the sedimentation rate (r).*

L143: this paragraph, and some others, are a mix of discussion and results and extracting the key message is therefore a bit difficult.

- *We split up this paragraph into two, enabling us to better emphasize the main point, which is that surveyed reservoirs have experienced most of the predicted sedimentation in 2025; we are confident in these results as they are based on observed data (lines 191-201).*

L215: have trapped?

- *Edited the text from “trap” to “have trapped.”*

L256: this sentence repeats the same message as the previous sentence?

- *Yes, we combined these two sentences to make the point more concise. Please see revised lines 280-283.*

L272: just to confirm, this would mean that (a part of) these 7% would then flow to the delta?

- *Yes, this 7% would have no remaining dams located below them, so this sediment would hypothetically flow freely to the coast.*

a bit broad comment: sections like L193, L292, L302, L315 feel a bit detached from your primary model results. Some of these sections don't really build on the (many) insights you can get from your model. At the same time, I can think of some others ideas that aren't discussed much (see major comment above).

- *We agree and removed the paragraph about the dam density and reservoir sedimentation comparison in the eastern versus the western US, since it is distracting from the main points of our article (original manuscript L193).*

- *One of our main goals of this paper is to highlight the need for agencies to consider sustainable sediment management in their reservoir operations, so this is why we discuss management options for terminal reservoirs, since targeting them would also potentially benefit downstream deltas (lines 314-320). We also want to highlight that sustainable sediment management also has great potential economic benefits, such as recovering lost water storage volume, as well as freeing up material for construction and restoration efforts (lines 321-329).*

- *We indeed expanded our discussion on sediment transport in the Introduction (lines 62-76). We also added discussion on potentially short time lags in sediment transport following dam removal to lines 310-313. We also plan to expand our discussion on the drivers of sedimentation in a future publication, which will modify the RATTES model to specific regions, where local climate and geology influences sedimentation patterns in varying ways.*

conclusion could be phrased more from the perspective of new scientific knowledge (and less about what the model can do?)

- *We revised the conclusion to include a summary of the major scientific knowledge gained and to emphasize the broader implications of our work. Please see lines 350-372.*

L543: a schematic figure of the model would be helpful here.

- *Thank you for this suggestion. We added a schematic figure of the model to the paper. Please see revised Figure 1.*

L650: A bit more information is needed to assess the uncertainty of the SYSR and SSUR models as I do not fully understand this. For example, uncertainty from a trap efficiency equation could be passed forward to RATTES uncertainty, right? Or uncertainty can be assessed by training the model on 2/3rds of the data and then applying it to the remaining 1/3rd?

- *Unfortunately, there is no error term available from the original Brown trap efficiency equation that we can carry through RATTES. We created uncertainty bounds, however, by re-running the models assuming all sediment retained is clay versus sand to illustrate how changing the k parameter changes the sedimentation and capacity results by modest percentages.*

- *We do in fact conduct the second suggestion by the reviewer, please see revised Methods lines 702-705.*

Reviewer #3 (Remarks to the Author):

What are the noteworthy results?

Leveraging a comprehensive database of 57,117 water-storage reservoirs across the continental United States, the authors quantify the volume of sediment trapped behind

dams and the associated loss of reservoir storage capacity. They translate this sediment sequestration into equivalent delta aggradation rates and evaluate whether these rates
could keep pace with eustatic and relative sea-level rise. The manuscript further provides estimates of the fraction of sediment stored behind terminal dams, highlighting a potential focus for sediment-management interventions.

Will the work be of significance to the field and related fields? How does it compare to the established literature? If the work is not original, please provide relevant references.
The United States is among the most heavily dammed regions globally. This study appears to be among the first high-resolution, contiguous US-wide, reservoir-by-reservoir estimates of sediment storage and associated capacity loss that explicitly includes small and mid-sized reservoirs. The findings are significant for quantifying anthropogenic alteration of sediment transfer from source to sink, especially in the context of climate-driven hydrologic change and accelerating sea-level rise. The results should be useful for river–delta restoration planning and for prioritizing sediment-management interventions and/or dam removal. More broadly, because sand and gravel are among the most extracted and economically important materials by volume, translating trapped sediment volumes into an approximate economic value (where feasible) could be particularly impactful for resource-management discussions.

Does the work support the conclusions and claims, or is additional evidence needed?
Conclusions and claims are generally supported by the data and results. A central assumption in estimating sediment volume is that reservoir capacity loss through time is an adequate proxy for sediment trapping and accumulation. It would strengthen the study to ground-truth this key assumption using lake-bottom cores from a subset of reservoirs with documented capacity surveys. Where available, such cores could provide a more direct constraint on sediment thickness (and accumulation rates) since dam construction and help evaluate potential biases from factors other than sedimentation (e.g., survey method changes, operational drawdown effects, or bathymetric uncertainty) that can also contribute to apparent capacity loss over time.

- *We appreciate this thoughtful suggestion. Please see the discussion above in the section “Responses to Major Comments” about how reservoir capacity loss reflects reservoir sedimentation. This assumption is well established and underpins the majority of reservoir sedimentation studies based on repeat bathymetric surveys. This approach has been widely applied across regional and national datasets[2,4,8,9], where capacity loss between surveys is interpreted as sediment storage within the reservoir.*

- *Direct validation using lake-bottom cores can indeed provide valuable local constraints on sediment thickness and accumulation rates. However, such data are rarely available at the spatial scale of this study, and existing national-scale sedimentation assessments similarly rely on repeat survey data rather than core-based measurements.*

Are there any flaws in the data analysis, interpretation and conclusions? Do these prohibit publication or require revision?
Data analysis and interpretation appear robust, and the conclusions are generally supported by the results. One issue that merits more explicit discussion is dam removal. In general, since the early 2000s the United States has been removing more dams than it is building. How does this trend affect (or potentially complicate) the authors' interpretation of reduced reservoir trap efficiency due to dam aging (lines 252–256)? In particular, do dam removals and decommissioning introduce a survivorship bias in the remaining reservoir population, and how is this accounted for in RATTES?

- *Please see the discussion above on dam removal in "Responses to Major Comments" in addition to the notes below. A key strength of our modeling framework is that dam construction and removal are explicitly represented through time within the reservoir network. In RATTES, both processes are incorporated on an annual basis, such that changes in upstream connectivity, sediment-contributing area, and storage capacity reflect the evolving state of the network. As a result, the reported capacity, sediment storage, and derived delta metrics inherently account for dam removal.*

- *For example, the Elwha River system illustrates this behavior (Figure 5B). The removal of Elwha Dam (2011–2012) and Glines Canyon Dam (2013–2014) leads to a stepwise reduction in upstream water storage and a corresponding decline in modeled reservoir sedimentation. Following complete removal, reservoir sedimentation ceases because no impoundments remain, and sediment is no longer retained within the former reservoirs. This is reflected in the abrupt termination of the Elwha trajectory in Figure 5B.*

- *Regarding the potential for survivorship bias, we emphasize that RATTES does not analyze a fixed population of reservoirs through time. Instead, it simulates a dynamically evolving network in which reservoirs enter and exit the system via construction and dam removal. Consequently, trends in trap efficiency reflect both 1) aging and infilling of remaining reservoirs and 2) structural changes in the network, including dam removal. To clarify this point, we have added text to the manuscript (lines 69-70 and 552) explicitly noting that observed trends integrate both aging effects and network restructuring, including dam removal.*

Is the methodology sound? Does the work meet the expected standards in your field?
The authors present a clear methodology, supported by detailed supplementary information, and key assumptions are stated throughout. Overall, the approach appears sound and consistent with expected standards in the field. One aspect that deserves more thorough treatment is the impact of sediment compaction. Although the authors briefly mention compaction (line 621), it should be explained in greater detail, including how compaction is represented in converting between deposited sediment volume, thickness, and mass. For example, if an exponential relationship between compaction and deposit age is assumed, the manuscript should justify the functional form and parameter choices, and discuss the sensitivity of results to these assumptions.

- *We appreciate this comment and agree that sediment compaction is an important process in reservoir sedimentation. In our framework, compaction is not explicitly modeled through a specific equation (e.g., exponential decay with deposit age) but is implicitly incorporated using repeat bathymetric surveys to constrain sedimentation rates. Because these rates are derived from differences between observed reservoir capacities over time, any compaction occurring between surveys is inherently included in the estimated net sediment accumulation. Including compaction effects is an additional reason why we collected data from only the first and last surveys as opposed to any intermediate surveys.*

- *We acknowledge that compaction can influence the relationship between deposited sediment volume, thickness, and mass, particularly over decadal timescales. In many reservoirs, sedimentation rates are highest in the years immediately following dam closure and subsequently decline as deposited sediments consolidate. As such, the time elapsed since dam closure is a key control on observed sediment volumes.*

- *To account for this effect, we include the parameter T (time since dam closure) in the model, which serves as an empirical proxy for time-dependent processes such as compaction. This is particularly important given that the median interval between repeat surveys in the training dataset (~35 years) is substantially shorter than the median elapsed time since dam closure for unsurveyed reservoirs used in prediction (approximately twice as long at 62 years).*

- *To evaluate the influence of this parameter, we re-trained the model without T, using only the three most important predictors (average trap efficiency, sediment-contributing drainage area, and specific discharge). Model performance remained comparable ($R^2 = 0.81$, RSE = 1.162) (Supplementary Table 1), but predicted total sediment storage in 2025 increased from 20.5 km^3 (with T) to 22.6 km^3 (without T). This result is consistent with expectations, as removing T reduces the model's ability to account for time-dependent reductions in apparent sedimentation rates associated with compaction.*

- *While we agree that a process-based treatment of compaction (e.g., explicit relationships between sediment age, porosity, and bulk density) would provide additional insight, such formulations require detailed stratigraphic or core data that are not available at the continental scale of this study. Accordingly, our approach follows standard practice in large-scale reservoir sedimentation assessments by relying on survey-derived capacity changes, with time since dam closure acting as an empirical proxy for unresolved processes such as compaction.*

- *We have revised the manuscript (lines 593-598, 694-698) to clarify how compaction is implicitly represented in the model, to better justify the role of the T parameter.*

- *More data would be needed to explicitly represent compaction based on grain size, accumulation rates and water level change.*

Is there enough detail provided in the methods for the work to be reproduced?
The authors provide sufficient methodological detail for the work to be reproduced. The code is available on GitHub, and the underlying data are provided in an Excel database, enabling others to replicate the analyses and results.

Suggestions for minor editing of the manuscript:
Line 11: Add the word "surface" after third word (i.e., "in surface reservoirs")
- *Reworded and included the word "surface" in line 13.*

Line 19: Clarify that this is total sediment (61.3 GT)
- *Added the word "total" in line 18.*

Line 29: Add the word "surface" after third word
- *Added the word "surface" in line 27.*

Line 59: Replace third word ("from") with "in previous"
- *Changed the wording as recommended.*

Line 333: Replace fourth word ("accurate") with "useful"
- *Changed the wording as recommended.*

Line 667: Replace "depth" with "thickness"
- *Changed the wording as recommended (line 749).*

Line 678-680: Check grammar. Sense is not clear.
- *We explained the sediment yield (SY) and statistical (MLR) model comparison in greater detail in the Supplementary Methods 1.*

References

1. *Dendy, F. M., Champion, W. A. & Wilson, R. B. Reservoir Sedimentation Surveys in the United States. in Man-Made Lakes: Their Problems and Environmental Effects 349–357 (American Geophysical Union (AGU), 1973). doi:10.1029/GM017p0349.*
2. *Minear, J. T. & Kondolf, G. M. Estimating reservoir sedimentation rates at large spatial and temporal scales: A case study of California. Water Resour. Res.* ***45****, (2009).*
3. *U.S. Army Corps of Engineers. Engineering and Design: Reservoir Water Quality Analysis. (1987).*
4. *Morris, G. L. & Fan, J. Reservoir Sedimentation Handbook : Design and Management of Dams, Reservoirs, and Watersheds for Sustainable Use. (McGraw-Hill, 1998).*
5. *Abeshu, G. W., Li, H.-Y., Zhu, Z., Tan, Z. & Leung, L. R. Median bed-material sediment particle size across rivers in the contiguous US. Earth Syst. Sci. Data* ***14****, 929–942 (2022).*
6. *Das, S., Talukdar, S. & Scaringi, G. Anthropogenic infrastructure overrides natural drivers of sediment yield in a major monsoonal river basin. Sci. Total Environ.* ***1010****, 181022 (2026).*
7. *Eckland, A. C. Reservoir Sedimentation and Delta Dynamics Across Scales. (University of Colorado at Boulder, United States -- Colorado, 2024).*
8. *Perera, D., Williams, S. & Smakhtin, V. Present and Future Losses of Storage in Large Reservoirs Due to Sedimentation: A Country-Wise Global Assessment. Sustainability* ***15****, 219 (2023).*
9. *Wisser, D., Frolking, S., Hagen, S. & Bierkens, M. F. P. Beyond peak reservoir storage? A global estimate of declining water storage capacity in large reservoirs. Water Resour. Res.* ***49****, 5732–5739 (2013).*
10. *Clow, D. W. et al. Organic Carbon Burial in Lakes and Reservoirs of the Conterminous United States. Environ. Sci. Technol.* ***49****, 7614–7622 (2015).*
11. *Texas Water Development Board. Completed Surveys & Data. https://www.twdb.texas.gov/surfacewater/surveys/completed/list/index.asp.*

12. *Bureau of Reclamation. RISE. https://data.usbr.gov/catalog?filter=generationEffortId.50.*
13. *Lane, E. W. & Koelzer, V. A. Density of Sediments Deposited in Reservoirs. (St. Paul U.S. Engineer District Sub-Office Hydraulic Laboratory, University of Iowa., 1943).*
14. *Miller, C. R. Determination of the Unit Weight of Sediment for Use in Sediment Volume Computations. (U.S. Department of the Interior, Bureau of Reclamation, Project Planning Division, 1953).*
15. *Syvitski, J. et al. Earth's sediment cycle during the Anthropocene. Nat. Rev. Earth Environ.* ***3****, 179–196 (2022).*
16. *Brown, C. Discussion, in Sedimentation in Reservoirs. Proc. Am. Soc. Civ. Eng.* ***69****, 1493–1500 (1944).*
17. *Brune, 1953. Trap efficiency of reservoirs. Eos Trans. Am. Geophys. Union* ***34****, 407–418 (1953).*
18. *Churchill, M. A. Discussion of 'Analysis and use of reservoir sedimentation data' by L. C. Gottschalk, pp. 139-140. in (Washington D.C., 1948).*
19. *Chen, C. N. Design of sediment retention basins. (1975).*
20. *Moragoda, N. et al. Modeling and Analysis of Sediment Trapping Efficiency of Large Dams Using Remote Sensing. Water Resour. Res.* ***59****, e2022WR033296 (2023).*
21. *Lehner, B. et al. High-resolution mapping of the world's reservoirs and dams for sustainable river-flow management. Front. Ecol. Environ.* ***9****, 494–502 (2011).*
22. *Steyaert, J. C., Condon, L. E., W. D. Turner, S. & Voisin, N. ResOpsUS, a dataset of historical reservoir operations in the contiguous United States. Sci. Data* ***9****, 34 (2022).*
23. *Hurst, A., Foster, M. & Eckland, A. The ResNet network of dams impounding storage reservoirs across the continental United States. Sci. Data (2025).*
24. *Prajapati, R., Gardner, J. & Prum, P. Extent of sediment concentration trends associated with climate and human factors across global rivers. Sci. Rep.* ***16****, 16062 (2026).*
25. *Tian, S. et al. Increasing river sediment concentration and flux across the pan-Arctic. Nat. Geosci.* ***19****, 565–572 (2026).*
26. *Dethier, E. N., Renshaw, C. E. & Magilligan, F. J. Rapid changes to global river suspended sediment flux by humans. Science* ***376****, 1447–1452 (2022).*

Responses to minor comments

Reviewer #1 (Remarks to the Author):

The authors consistently modified the manuscript according to the three reviewers' comments.
The rebuttal gives responses to major comments as well as to individual reviews.
I highly appreciate the endurance and clarity of the response, and modifications and additions in the manuscript's text and figures.
I have no objection for publication at this stage.

- *Thank you for your constructive review!*

Reviewer #2 (Remarks to the Author):

Review by Jaap Nienhuis.

I have now re-read the manuscript and I think it has been greatly improved. I thank the authors for their answers to my earlier review and I have only a few remaining remarks:

- *Thank you for your constructive review!*

One thing I would like to clarify about my earlier review, reading the author's subsequent response, is that I realize that the RATTES model does not explicitly resolve fluvial sediment transport. Nevertheless, the presented results still would benefit from the context of (spatial/temporal) observations of the effect of dams on sediment fluxes to deltas, especially if implications are drawn for delta resiliency. For example: the authors present a sediment flux (sediment volume over time) trapped by dams in figure 3a, and further present several hints about temporal aspects of sediment trapping (Fig 5b). How does this flux compare to observations of sediment flux increase across North American rivers reported by Dethier (2022), or the Mississippi work by Nittrouer & Viparelli (2012)? Is there any relation between the (predicted) sediment storage in dams and the (observed) sediment flux increase to deltas? Such a comparison (perhaps only qualitatively in the discussion) would provide significant strength (or necessary nuance) to your conclusions about delta resilience.

- *Thank you very much for this helpful suggestion. We agree that placing our modeled sediment trapping within the context of observed sediment fluxes to coastal deltas provides important perspective, particularly given our discussion of delta resilience. As the reviewer notes, RATTES does not explicitly simulate fluvial sediment transport through river networks or sediment delivery to the coast. Rather, it estimates sediment yield and storage within the dammed portion of each watershed through time (Figure 1). Consequently, direct comparisons with observed coastal sediment fluxes must be interpreted with caution.*
- *To address this comment, we added two paragraphs to the end of the section “Upstream sediment trapping limits potential delta aggradation” (Results, lines 266-290), together with new Supplementary Data 3. These additions compare our modeled sediment yields with*

historical and modern sediment flux observations for major North American rivers such as the Mississippi and Colorado. To facilitate a first-order comparison with observed coastal sediment fluxes, we extrapolate the modeled sediment yield from the dammed portion of each basin to the full upstream drainage area, while explicitly noting the assumptions and limitations of this approach. We show that these extrapolated values are generally within the same order of magnitude as published observations, while also explaining why they often exceed measured coastal fluxes due to the well-established decrease in sediment yield with increasing drainage area and the fact that RATTES estimates sediment yield rather than sediment delivery to oceans.

- *We also expanded the discussion to place these comparisons in the context of previous studies, including Dethier (2022) and Nittrouer & Viparelli (2014). Together, these additions emphasize that substantial sediment production may persist within many watersheds even where dams and flow regulation substantially reduce sediment delivery to the coast, providing additional context and nuance for our conclusions regarding reduced delta resilience.*

minor comments:
L130: it would be useful here to say explicitly which (anthropogenic) parameters are important. The formula (1) only has geomorphological/hydrological characteristics (drainage area, specific discharge), but this is probably not what you mean.

- *Thank you, great point. We add a sentence to expand and clarify (lines 124-127). We include the relative contribution of each parameter in explaining the total model variance. The sediment-contributing drainage area (A_{sedMLR}), which is the total drainage area reduced by the dammed drainage area, explains 66.8% of the total variance in R, while time between reservoir surveys (T) (a proxy for dam age) explains 7.2% of the variance, dam trap efficiency (T_E) explains 7.1%, and specific discharge (S) explains 0.2%. We characterize A_{sedMLR}, T, and T_E as anthropogenic parameters because they all pertain to dams themselves, while S is solely a geomorphologic characteristic comprising information about runoff and total drainage area.*

L174: I find this a very important conclusion of your study. Perhaps highlight it more in the abstract/conclusion?

- *Thank you for your comment. We agree that this is an important conclusion of our study. However, we do not want to over-emphasize this point because the total capacity loss nationwide depends heavily on the original design capacity studied. Overall, we predict similar sedimentation rates with other studies but less capacity loss because of our inclusion of smaller dams and incorporation of changing trap efficiency. We also do not assume constant sedimentation, which we have highlighted. Please see lines 162-165 where we re-emphasize these points.*

Reviewer #3 (Remarks to the Author):

I thank the authors for their careful and detailed responses to my comments and other reviewers'.

Overall, I am satisfied with the level of effort in addressing the reviewers' comments and the amount of revision. The additional text clarifying the methods and underlying concepts will help readers understand the manuscript, and the new schematic in Fig. 1 is helpful for conveying the modeling approach, the governing equations, and the analyses. My three principal concerns from the first round were each engaged directly, as summarized below.

- *Thank you for your constructive review!*

1. Reservoir capacity loss as a proxy for sediment accumulation
The authors have strengthened the justification for this central assumption (lines 588-598), citing bathymetric reports that document rising reservoir-bottom elevations over time, together with Bureau of Reclamation cores and Texas Water Development Board geophysical surveys at selected sites. I appreciate also the added screening of the survey data (removing records where capacity increased and requiring a minimum 10-year inter-survey interval), which addresses part of the survey-method and dam-raise artifacts I raised. This is a reasonable and field-standard treatment, and I accept the authors' point that a quantitative, core-based ground-truth is not feasible at the national scale of this study.

One suggestion: if at all possible, it would meaningfully strengthen reader confidence if the authors could show, in the supplementary material, at least one example sediment core paired with the corresponding capacity-loss survey at the same reservoir. Even a single well-documented case (e.g., a Bureau of Reclamation site) presented as a supplementary figure would directly illustrate that measured capacity loss corresponds to accumulated sediment thickness. I leave this to the authors' discretion given data availability.

  - *Thank you for this helpful comment. We added Supplementary Note 4, which details some examples of sediment cores coupled with bathymetric surveys and points the reader to figures within the applicable reports. Given our timeline, we did not seek permission to reproduce these figures in the supplemental. We highlight figures included within the Texas Water Development Board's Lake Travis Survey Report and the papers published detailing sediment cores, geophysical surveys, and the bathymetric survey at Lake Englebright in California.*

2. Dam removal and survivorship bias
This is addressed well. The author clarify that RATTES is not an analysis of a fixed reservoir population but a dynamically evolving network in which reservoirs enter (construction) and exit (removal) on an annual basis, with the sediment-contributing drainage area re-adjusted for downstream structures after each removal (lines 69-70, 552). This design directly defuses my survivorship-bias concern, since the model simulates the actual time-evolving population rather than filtering to surviving reservoirs. The Elwha example (Figure 5B) is a clear illustration, and the context that ~2,350 documented removals are small relative to >57,000 standing dams is helpful. I also appreciate the authors' take about what the volumetric framework cannot do: namely, route the sediment pulse released downstream following a removal. That limitation is appropriately stated and is acceptable for the scope of this paper.

- *Thank you.*

3. Sediment compaction

The treatment is improved and the response is made in good faith. The authors explain that compaction is captured implicitly because sedimentation rates are derived by differencing repeat bathymetric surveys, and they have forced the time parameter (T) into the model as an empirical proxy for time-dependent processes including compaction, supported by a sensitivity test in which removing T changes the predicted volume by roughly 10% while R^2 remains stable (lines 593-598, 694-698). I accept the argument that an explicit compaction function is not supportable at the national scale.

Two small points would further tighten this section. First, the single reported mass (61.3 Gt), which underpins the prominent comparison that US reservoir sediment exceeds the annual global fluvial load by roughly seven times, rests on a single constant bulk density (960 kg/m^3). Because density varies with sediment age and grain size (the very compaction effect under discussion), I recommend the authors attach a brief sensitivity range to that mass figure so the headline comparison carries an explicit uncertainty. Second, the sensitivity values cited in the rebuttal (predicted storage changing from 20.5 to 22.6 km^3 when T is removed) do not obviously reconcile with the 63.8 km^3 total reported in the main text; if these refer only to the unsurveyed/MLR component, a one-line clarification in the text or supplement would avoid confusion.

- *Thank you for these suggestions. We add a mass range for US reservoir sedimentation, from 44.4 to 103.7 GT, and report this in the abstract (lines 18-19) and main text (lines 138-139). We calculate this range by taking the lower and upper bounds for total US sedimentation (60.2 to 69.6 km^3) derived from the additional clay and sand runs, and converting these values to masses using the dry bulk density for clay and sand, respectively. We used a clay bulk density of 736.8 kg/m^3 and sand bulk density of 1489.7 kg/m^3 from Table 10, Lane and Koelzer (1943), which summarizes bulk densities for reservoir sediments based on operations (i.e., water level variability) after one year. We used the values from Scenario B, where the operation typically undergoes moderate reservoir drawdown. These mass sedimentation rates are conservative since bulk density typically increases as sediment compacts over many years.*
- *We add the result of the sensitivity analysis to the main text in lines 133-135, stating that by removing the time parameter, T, we predict a larger stored sediment volume since the effects of compaction are not implicitly included.*

Minor Points

All seven of my first-round minor edit have been incorporated.
